# Picorna-Like Viruses of the Havel River, Germany

**DOI:** 10.3389/fmicb.2022.865287

**Published:** 2022-04-04

**Authors:** Roland Zell, Marco Groth, Lukas Selinka, Hans-Christoph Selinka

**Affiliations:** ^1^Section of Experimental Virology, Institute for Medical Microbiology, Jena University Hospital, Friedrich Schiller University, Jena, Germany; ^2^CF DNA Sequencing, Leibniz Institute on Aging, Fritz Lipmann Institute, Jena, Germany; ^3^Section II 1.4 Microbiological Risks, Department of Environmental Hygiene, German Environment Agency, Berlin, Germany

**Keywords:** riverine ecosystems, viromes, *Picornavirales*, phylogenetic analysis, metagenomic, picorna-like viruses, RNA viruses

## Abstract

To improve the understanding of the virome diversity of riverine ecosystems in metropolitan areas, a metagenome analysis was performed with water collected in June 2018 from the river Havel in Berlin, Germany. After enrichment of virus particles and RNA extraction, paired-end Illumina sequencing was conducted and assignment to virus groups and families was performed. This paper focuses on picorna-like viruses, the most diverse and abundant group of viruses with impact on human, animal, and environmental health. Here, we describe altogether 166 viral sequences ranging in size from 1 to 11.5 kb. The 71 almost complete genomes are comprised of one candidate iflavirus, one picornavirus, two polycipiviruses, 27 marnaviruses, 27 dicistro-like viruses, and 13 untypeable viruses. Many partial picorna-like virus sequences up to 10.2 kb were also investigated. The sequences of the Havel picorna-like viruses represent genomes of seven of eight so far known *Picornavirales* families. Detection of numerous distantly related dicistroviruses suggests the existence of additional, yet unexplored virus groups with dicistronic genomes, including few viruses with unusual genome layout. Of special interest is a clade of dicistronic viruses with capsid protein-encoding sequences at the 5′-end of the genome. Also, monocistronic viruses with similarity of their polymerase and capsid proteins to those of dicistroviruses are interesting. A second protein with NTP-binding site present in the polyprotein of solinviviruses and related viruses needs further attention. The results underline the importance to study the viromes of fluvial ecosystems. So far acknowledged marnaviruses have been isolated from marine organisms. However, the present study and available sequence data suggest that rivers and limnic habitats are relevant ecosystems with circulation of marnaviruses as well as a plethora of unknown picorna-like viruses.

## Introduction

The order *Picornavirales* is comprised of eight families, *Caliciviridae*, *Dicistroviridae*, *Iflaviridae*, *Marnaviridae*, *Picornaviridae*, *Polycipiviridae*, *Secoviridae*, and *Solinviviridae* with so far 100 genera and 323 species (as of 5 December 2021).^1^ Viruses of this order have a single-stranded RNA genome with positive polarity and an icosahedral capsid with *T* = 3 or *T* = 1/pseudo T3 structures (Le Gall et al., 2008). The *T* = 1/pseudo *T* = 3 capsids of the *Picornavirales* members consist of 60 copies of three major capsid proteins (CP) with a characteristic jelly roll fold; an additional minor capsid protein (VP4) may be present (Rossmann and Johnson, 1989). As an exception, some viruses of the *Secoviridae* family with *T* = 1/pseudo T3 structure have either only one large CP with three jelly roll domains, or two CPs, a small CP with one jelly roll domain and a large CP with two (Chen et al., 1987; Chandrasekar and Johnson, 1998). Caliciviruses and solinviviruses have a *T* = 3 capsid which consists of 180 copies of a single CP (Prasad et al., 1999; Valles et al., 2014). Viral RNAs of all members of the *Picornavirales* are polyadenylated but exhibit a considerable variation of their genome organization, e.g., monopartite or dipartite genomes which encode one to five open reading frames (ORFs). The ORFs are expressed by various mechanisms including the usage of one or two internal ribosome entry sites (*Picornaviridae*, *Dicistroviridae, Iflaviridae, Marnaviridae, and Polycipiviridae*), ribosomal frame-shifting (Solenopsis invicta virus 3 of the *Solinviviridae*), transcription of subgenomic RNAs (*Caliciviridae, Labyrnavirus, and Solinviviridae*), or reinitiation (*Caliciviridae and Polycipiviridae*; Jan, 2006; Valles et al., 2014; Zinoviev et al., 2015; Olendraite et al., 2017). Despite all differences in genome layouts, all members of the *Picornavirales* and many yet unclassified picorna-like viruses (PLVs) share in common a set of phylogenetically related proteins, especially (i) a helicase (hel) with one or two conserved sequence motifs of P-loop ATPases (Walker A and B motifs), (ii) the chymotrypsin-like proteinase (pro) with a CxCG or CxSG active site sequence motif, (iii) the RNA-dependent RNA polymerase (RdRP or pol) with conserved SG, GDD, and LK sequence motifs, and (iv) one to three CPs with a jelly roll fold. A short genome-linked viral peptide (VPg) encoded by a gene region located between the hel and pro genes has been shown for six of the eight families. Polycipiviruses and solinviviruses are expected to possess a VPg as well, although an experimental proof is still lacking (Olendraite et al., 2017; Brown et al., 2019). The hel-pro-pol domains in this order are known as “hel-pro-pol core replicative module” or “hel-pro-pol replication block” (Le Gall et al., 2008; Sanfaçon et al., 2012). The CPs are encoded either (i) together with the nonstructural proteins (NSP) as part of a large polyprotein (e.g., *Iflaviridae*, most members of the *Picornaviridae*, and many members of the *Marnaviridae*), (ii) by a second ORF (e.g., *Dicistroviridae*, many viruses of the *Marnaviridae*, and dicipiviruses of the *Picornaviridae*), (iii) a subgenomic RNA (*Caliciviridae*, *Labyrnavirus* of the *Marnaviridae*, and *Solinviviridae*), (iv) a second RNA molecule (some members of the *Secoviridae*), or (v) overlapping ORFs expressed by ribosomal frame-shifting (*Solinviviridae*; for references, see Valles et al., 2007, 2014; Bonning and Miller, 2010; van der Vlugt et al., 2015; Thompson et al., 2017; Zell, 2018). In addition, genomes may show family-specific and genus-specific gene regions encoding conserved or unique proteins, like the leader protein of iflaviruses or many picornaviruses with various functions, the movement protein of some secoviruses, or the ovarian tumor domain of solinviviruses. The order of CP- and NSP-encoding gene regions may vary: the monocistronic iflaviruses, picornaviruses, and some secoviruses have large polyproteins with CP domains at the N-terminus and NSP domains at the C-terminus; polycipiviruses have CP-encoding ORFs at the 5′-end of their genomes, whereas caliciviruses, dicistroviruses, marnaviruses, and solinviviruses encode these proteins at the 3′-end of their genomes. Also, the position of the VP4, a minor CP of some *Picornavirales* families, may vary. Viruses of the *Picornavirales* infect a wide range of eukaryotic organisms. Hosts of six vertebrate classes are infected by picornaviruses, mammals, and fish by caliciviruses (Zell, 2018; Smertina et al., 2021), whereas dicistroviruses, iflaviruses, polycipiviruses, and solinviviruses are associated with arthropods (Valles et al., 2017a,b; Brown et al., 2019; Olendraite et al., 2019). Secoviruses infect (dicotyledonous) plants and marnaviruses diatoms, unicellular algae, and heterotrophic protists (Sanfaçon et al., 2009; Vlok et al., 2019).

It is long known that virioplankton, i.e., free-floating viruses in aquatic ecosystems, is quite ubiquitous, outnumber microbes and other organisms many times over and are important players in maintaining stable food webs and nutrient cycles (Fuhrman, 1999; Wilhelm and Suttle, 1999; Wommack and Colwell, 2000; Culley et al., 2003). Development of next-generation sequencing techniques and metagenomics pushed forward the description of a plethora of novel viruses infecting prokaryotes, protists, animals, and plants in marine, freshwater and terrestrial ecosystems which advanced our view on the virosphere (e.g., Culley and Steward, 2007; Rosario and Breitbart, 2011; Culley et al., 2014; Wommack et al., 2015; Fierer, 2017; Williamson et al., 2017; Coy et al., 2018; Lefeuvre et al., 2019). In particular, presence of PLVs in environmental samples indicates their abundant prevalence in marine ecosystems (Culley and Steward, 2007; Culley et al., 2014; Lang et al., 2018). However, culture-independent virus sequencing enabled also the identification of numerous PLVs in fecal and organ samples of many unexpected organisms indicating both wide distribution in organismic kingdoms but also suggesting unspecific virus uptake and accumulation without subsequent infection (e.g., Shi et al., 2016, 2018; Yinda et al., 2017; Dastjerdi et al., 2021).

In the present study, we demonstrate the presence of 166 mostly novel PLVs in the river Havel, designated Havel picorna-like virus (HPLV) 1 to −166. Seventy-one almost complete genomes (i.e., complete coding regions plus parts of the 5′- and 3′-untranslated regions) and many partial genomes of seven of eight families of the *Picornavirales* demonstrate a remarkable genetic diversity of this virus order in a riverine ecosystem whose overall complexity is still unexplored.

## Materials and Methods

### Sample Collection and Virus Enrichment

A freshwater sample with a volume of 50 liters was collected from the river Havel in the metropolitan area of Berlin, Germany, on June 28th, 2018 (sampling site coordinates 52°30′46″N13°12′14″E). The sample was transported under cooled conditions to the laboratory and immediately processed. Five 10 L samples were homogenized by vortexing (20 min) and preconditioned for enhanced virus binding to negatively charged glass wool by adjusting the pH from 8.1 to 3.5. Thereafter, virus particles were concentrated by glass wool filtration as basically described by Wyn-Jones et al. (2011). Virus concentrates were slowly eluted from the column with a buffer (pH 9.5) containing 3% (w/v) beef extract in 0.05 M glycine and subsequently adjusted to pH 7.0 without further flocculation. The 180 ml eluate was filtered through a 0.45 μm filter to remove bacteria and detritus. Then, virus particles of the filtrate were sedimented by ultracentrifugation at 100,000 × *g* for 2.5 h at 4°C. Sediment was resolved in a total of 500 μl PBS and homogenized using a ball mill. RNA was extracted using the QIAamp Viral RNA mini kit (Qiagen, Hilden, Germany). One hundred nanogram RNA was employed for library preparation.

### Virus Sequencing and Sequence Data Processing

The Illumina next-generation sequencing approach was used in our study (Bentley et al., 2008). The library was prepared from 100 ng of RNA using the TruSeq stranded mRNA library preparation kit (Illumina). In order to address all RNA molecules (not only polyadenylated RNA), the protocol was adapted as follows: RNA was precipitated using isopropanol and resolved in *Fragment, Prime, Finish Mix* (FPF). From this step on, the manufacturer’s protocol was followed (p20, step 12, TruSeq Stranded mRNA Sample Preparation Guide, Part # 15031047 Rev. E, Illumina). The obtained library was quantified and quality-checked using the Agilent 2100 Bioanalyzer and the DNA 7500 kit (Agilent Technologies). Sequencing was done on a HiSeq 2500 running in rapid-mode, paired-end (2× 150 bp) by combining one 200 cycle and two 50 cycle SBS kits (Illumina, FC-402-4021 and FC-402-4022). Sequence data were extracted in FastQ format using Illumina’s tool bcl2FastQ v2.19.1.403.

Reads were pre-processed before assembly. Adapter and quality trimming were done using cutadapt v1.8.3 (Martin, 2011); parameters: -q 10 -m 30 -a AGATCGGAAGAGCACACGTCTGAACTCCAGTCA -A AGATCGGAAGAGCGTCGTGTAGGGAAAGAGTGT. Then, amplification duplicons were removed by comparing the sequence of all pairs against each other. In the next step, read pairs were assembled using two different software tools, metaSPAdes v3.15.3 (Nurk et al., 2017) with standard parameters (-k auto) and the clc_assembler v. 5.2.1 (part of the CLC Assembly Cell, Qiagen; parameters: -p fb ss 0850).

### Sequence Data Analysis

The contigs, as result from both assembly tools, were used to search a protein database created with all NCBI GenBank entries for the Taxonomy ID 10239 (search term “viruses[organism]”) utilizing DIAMOND (Buchfink et al., 2015) and BLAST+ version 2.6.0,^2^ respectively. In parallel, contigs were compared with 12 reference data sets consisting of reference sequences of the families and sub-families of the *Picornavirales* order using tBLASTx. Then, translated candidate sequences with significant similarity to reference sequences were queried against the contig bank to identify contigs with similar sequences. Virus contigs were manually curated to generate the final full-length and partial genomes greater 1 kb. Protein domains were predicted using the Pfam conserved domain database (CDD) search tool of NCBI.^3^

For phylogenetic analyses, protein sequences of the present study and reference sequences of the GenBank were aligned with Mega version X (Kumar et al., 2018) and adjusted manually. Maximum likelihood trees were inferred with IQ-TREE 2.1.3 for Windows (Nguyen et al., 2015). Branch supports were assessed with UFBoot2 implemented in IQ-TREE software (Hoang et al., 2018). Usually, 50,000 ultrafast bootstrap replications (-B 50000) were conducted. Best-fitting nucleotide substitution models (-m MFP) were selected on basis of the Bayesian Information Criterion (BIC) with ModelFinder also implemented in IQ-TREE.

## Results

### High-Throughput Sequencing, Assembly, and Identification of Viral Sequences

A 50-liter freshwater sample of the river Havel was used for virus enrichment, RNA extraction, and paired-end sequencing on an Illumina HiSeq 2,500 platform. Sequencing yielded a total of 144,395,487 read pairs. After removal of duplicons, 51,902,006 read pairs were obtained and utilized for assembly. The assembly process resulted in (i) metaSPAdes: 484,430 scaffolds (total length 132,507,247 nt; N50: 272 nt) and (ii) CLC assembler: 162,082 contigs (total length: 62,667,741 bp; N50: 382 bp). DIAMOND assigned 5,687 scaffolds (1.17%) and 3,902 CLC contigs (2.4%), respectively, to the *Picornavirales* order, but only 2,453 (41.6%) and 1,612 (41.3%), respectively, could be classified into one of the eight *Picornavirales* families. In a parallel approach, contigs with similarity to viruses of the *Picornavirales* (>100 identical amino acids) were identified by BLAST searches against 12 data sets of representative sequences of each of the *Picornavirales* families and sub-families. In this study, we further analyzed a total of 166 sequences with lengths greater 1 kb of which 71 sequences represented an almost complete virus genome (Supplementary Table 1).

### *Dicistroviridae* and *Marnaviridae*

**Figure 1:**
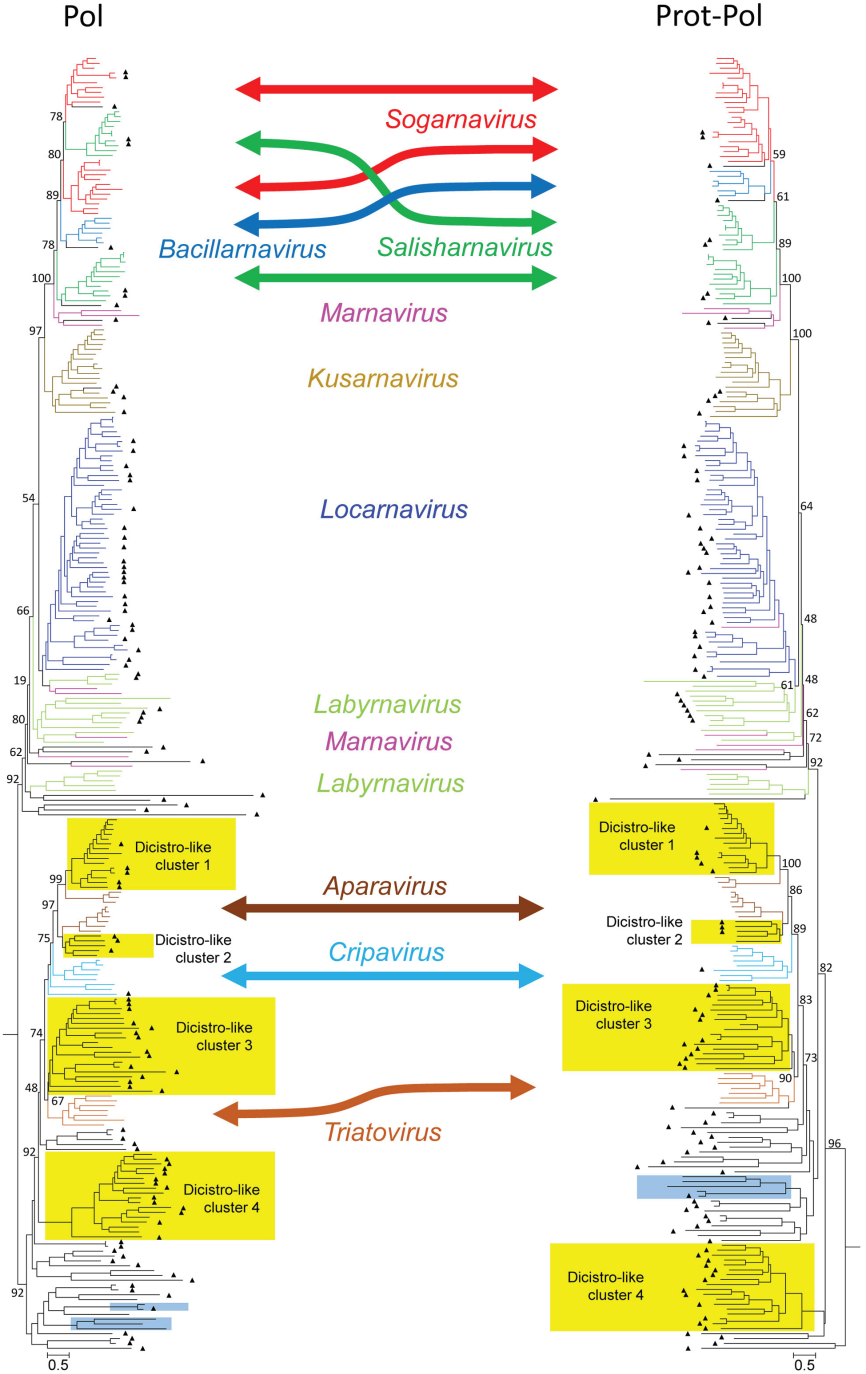
Phylogenetic analysis of 267 polymerase- (left) and 264 proteinase/polymerase-encoding sequences (right) of dicistroviruses, marnaviruses, and unassigned viruses. Sequences were aligned with MEGA and manually adjusted. The trees were inferred with IQ-Tree 2, optimal substitution model: GTR + F + R9 for the pol tree and GTR + F + R10 for the prot/pol tree, respectively. Numbers at nodes present bootstrap values obtained after 50,000 ultrafast bootstrap replications. The scale indicates substitutions per site. The respective genera are indicated. Color codes: aparaviruses, dark brown; bacillarnaviruses, blue; cripaviruses, light blue; kusarnaviruses, ochre; labyrnaviruses, light green; locarnaviruses, dark blue; marnaviruses, magenta; salisharnaviruses, dark green; sogarnaviruses, red; triatoviruses, brown; and untypeable viruses, black. A triangle (▲) indicates viruses identified in the present study. Blue boxes indicate viruses with unusual genome layout (capsid protein-encoding gene region at the 5′-end; nonstructural polyprotein-encoding gene region at the 3′-end). Yellow boxes indicate four dicistrovirus-like sequence clusters. More details of this figure are presented in Supplementary Figure 1.

The dominant PLVs detected in the Havel river can be classified as dicistrovirus- and marnavirus-like as judged from preliminary phylogenetic analyses of the polymerase and helicase gene regions (data not shown). Eighty-five percent of the identified HPLV sequences (141/166) clustered with sequences of viruses of these families. For a further analysis, 109 HPLV polymerase sequences were aligned with 35 reference sequences representing all 10 genera of both families as well as 120 unclassified viruses with similarity to our HPLVs (Figure 1, Supplementary Figure 1). The phylogenetic tree reveals two major branches with monophyletic clades corresponding to the genera *Bacillarnavirus*, *Kusarnavirus*, *Locarnavirus*, and *Triatovirus*. The remaining genera *Aparavirus*, *Cripavirus*, *Labyrnavirus*, *Marnavirus*, *Salisharnavirus*, and *Sogarnavirus* were not monophyletic. In addition, several clades of HPLVs and other unclassified viruses clustered on both major branches with various bootstrap values. Long maximum likelihood distances indicated substitution saturation of some sequences. In order to improve the robustness of the tree, a phylogenetic analysis was conducted with an alignment of the proteinase/polymerase sequences of these viruses. The topology of the resulting tree presented similar to that of the polymerase tree. Interestingly, in contrast to the polyphyletic sequences of sogarnaviruses and cripaviruses based on the polymerase gene alone, sequences of both genera were monophyletic by analyzing the proteinase/polymerase sequences together (Figure 1, Supplementary Figure 1). Altogether, the tree reveals one bacillarna-like, two marna-like, four kusarna-like, three sogarna-like, four salisharna-like, six labyrna-like, and 23 locarna-like HPLVs on the marnavirus branch. The newly identified HPLV-20 clustered with kusarnaviruses in both trees but exhibited an unusual genome layout: the CP-encoding gene region was located at the 5′-end of the genome, the NSP-encoding gene region at the 3′-end. Comparison of the polymerase and proteinase/polymerase trees revealed only few HPLV strains with inconsistent clustering (e.g., HPLV-10; HPLV-91).

Moreover, as also shown in Figure 1 (Supplementary Figure 1), the dicistrovirus branch of both trees was comprised of the three genera *Aparavirus*, *Cripavirus*, and *Triatovirus* plus several clades of unclassified viruses with both dicistronic and monocistronic genome layouts. Only HPLV-102 was identified as a dicistrovirus candidate on basis of the proteinase/polymerase sequence. However, many short contigs were identified with strong similarity to members of the acknowledged dicistroviruses (data not shown). The dicistrovirus-like clades 1 and 2 consistently clustered with high bootstrap support with aparaviruses and may represent new aparavirus species. Dicistrovirus-like clade 3 clusters with triatoviruses, whereas dicistrovirus-like clade 4 is less well supported. Both clades include altogether 28 sequences, but only three viruses were obtained from freshwater arthropods (KX883663, KX883640, and KX883650); the remaining viruses were detected in samples as diverse as Havel river water, grassland soil, fecal specimens/intestinal contents of mammals, cloacal swabs of birds, higher plants, freshwater snails, or marine oysters. Several viruses have been proposed as novel dicistroviruses previously (e.g., Reuter et al., 2014; Yinda et al., 2017; Duraisamy et al., 2018; Dastjerdi et al., 2021). Three clades contain viruses with monocistronic genomes. Also of interest are HPLV-32 and -150 with their unusual genome layout (CP-encoding gene region at the 5′-end; NSP-encoding gene region at the 3′-end).

As marnaviruses and dicistroviruses share a remarkable similarity of the structural proteins (Lang et al., 2018), we also conducted a phylogenetic analysis of the CPs and largely confirmed the major clades seen with the polymerase and proteinase/polymerase gene region (see Supplementary Figure 2). The CP tree revealed a second cripavirus candidate from the Havel river (HPLV-141), but also few viruses which displayed inconsistent clustering (e.g., HPLV-20, −38, −40, −52, −61, and −91).

### Caliciviridae

**Figure 2:**
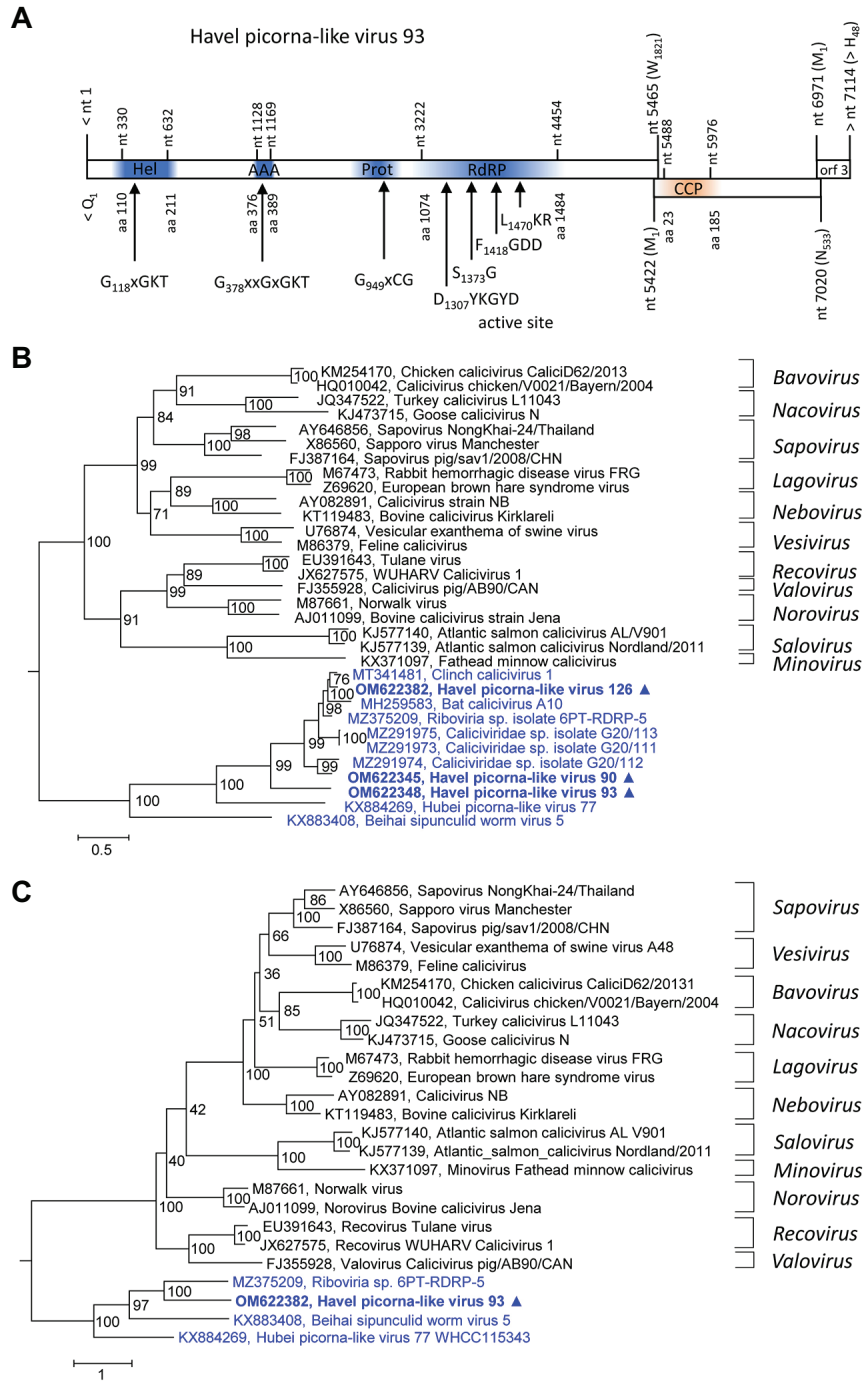
Analysis of new caliciviruses. **(A)** The genome organization of Havel picorna-like virus 93 resembles the calicivirus genome. ORF1 shows conserved elements of helicase (Hel), proteinase (Prot), and polymerase (RdRP) domains, whereas ORF2 has similarity to the calicivirus capsid protein (CCP) family of caliciviruses. Conserved domains and sequence motifs were identified by searching the NCBI Conserved Domain Database (https://www.ncbi.nlm.nih.gov/Structure/cdd/wrpsb.cgi). Phylogenetic analyses of the VP1 (capsid protein) gene region **(B)** and the proteinase/polymerase gene region **(C)** of calicivirus prototype strains, unassigned caliciviruses, and Havel picorna-like viruses 93, −175, and −176 are shown. The trees were inferred with IQ-Tree 2, optimal substitution model: TVM + F + R4 **(B)** and TVMe+R4 **(C)**. Numbers at nodes present bootstrap values obtained after 50,000 ultrafast bootstrap replications. The scale indicates substitutions per site. Presented are GenBank acc. nos. and virus names. The respective genera are indicated to the right. Unassigned viruses are printed in blue. A triangle (▲) indicates the viruses of the present study. AAA, AAA protein with homology to ATPase family associated with various cellular functions, Hel, helicase, Prot, proteinase; RdRP, polymerase; and CCP, capsid protein with similarity to the calicivirus capsid protein.

Three new caliciviruses were identified. The partial genome of HPLV-93 corresponds to about 90% of a typical calicivirus genome and contains three partly overlapping ORFs. The available sequence comprises the almost complete NSP-encoding gene region of ORF1 [1821 amino acids (aa)], the complete ORF2 (533 aa) with the calicivirus coat protein (CCP) gene region, and a partial ORF3 region (48 aa). The NSP precursor comprises a helicase domain (modified Walker A motif: G_118_xGKT), a second Walker A motif at aa position 378, a proteinase domain with a G_949_xCG active site motif, and an RdRP which shows the conserved D_1307_xxxxD, S_1373_G, F_1418_GDD, and L_1470_KR sequence motifs (Figure 2A). Two other partial genomes of new caliciviruses, HPLV-90 (2,748 nt) and −126 (1,038 nt), contain polymerase to VP1 and VP1 sequences, respectively. Phylogenetic analysis of the CCP-encoding gene region (VP1) of these HPLVs suggests novel caliciviruses which show similarity to the classified caliciviruses but are more closely related to unclassified caliciviruses from bats, lizards, insects, annelids, and various bivalve shellfish (Figure 2B). This result is confirmed by analysis of the proteinase/polymerase gene region (Figure 2C). Genetic distances of the VP1 protein in pairwise comparisons with other caliciviruses reveal values greater 74%, suggesting that they belong to a new genus of the *Caliciviridae* family (Supplementary Table 2).

### Iflaviridae

**Figure 3:**
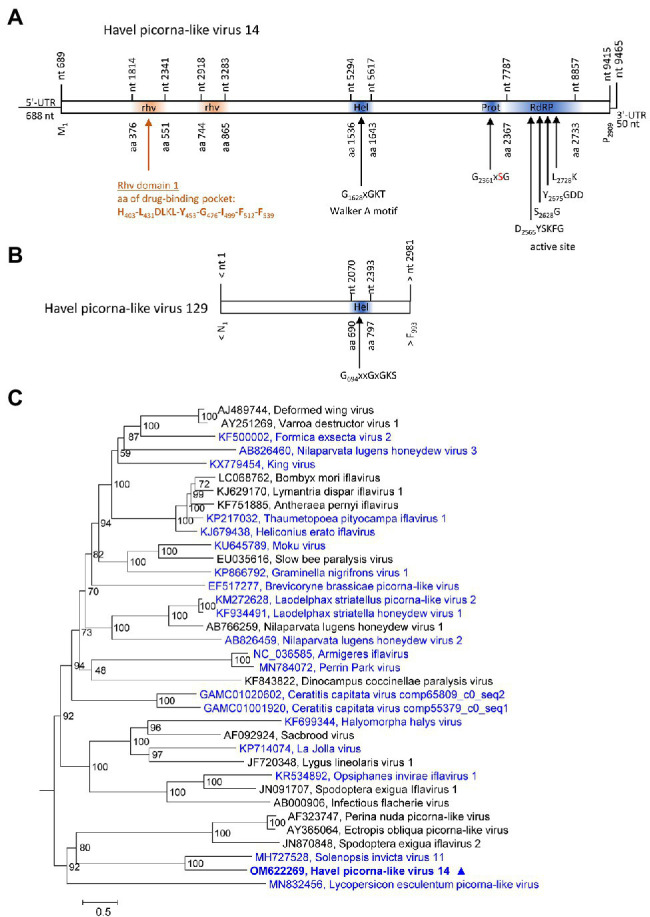
Analysis of Havel picorna-like viruses 14 and 129. Their genome organization **(A,B)** corresponds to the iflavirus genome. Conserved domains and sequence motifs were identified by searching the NCBI Conserved Domain Database (https://www.ncbi.nlm.nih.gov/Structure/cdd/wrpsb.cgi). **(C)** Phylogenetic analysis of the complete ORF of 15 acknowledged iflaviruses and 21 candidate strains including Havel picorna-like virus 14 and 129. The tree was inferred with IQ-Tree 2, optimal substitution model: GTR + F + R5. Numbers at nodes present bootstrap values obtained after 50,000 bootstrap replications. The scale indicates substitutions per site. Presented are GenBank acc. nos. and virus names. Unassigned viruses are printed in blue. A triangle (▲) indicates the virus of the present study. UTR, untranslated region; Hel, helicase; Prot, proteinase; RdRP, polymerase; and rhv, capsid protein with similarity to the rhinovirus capsid protein with jelly roll fold and drug-binding pocket.

Two contigs representing HPLVs with similarity to iflaviruses were investigated. Iflaviruses have a monocistronic genome encoding a large polyprotein with a leader protein and CP domains at the N-terminus and NSPs at the C-terminus. HPLV-14 (9,465 nt) has an almost complete genome with iflavirus gene layout, whereas the partial genome of HPLV-129 (3,028 nt) contains the helicase gene region only (Figures 3A,B). For the phylogenetic analysis shown in Figure 3C, aligned ORF sequences of 36 acknowledged and unclassified iflaviruses were investigated. The phylogenetic tree indicates closest similarity of HPLV-14 to solenopsis invicta virus 11. A second phylogenetic analysis based on partial sequences (VP1 to helicase gene region), which included both the HPLV-14 and HPLV-127 sequences, confirmed relationship of both HPLVs to the classified iflaviruses. Both viruses may belong to different novel iflavirus species (Supplementary Figure 3).

### Picornaviridae

**Figure 4:**
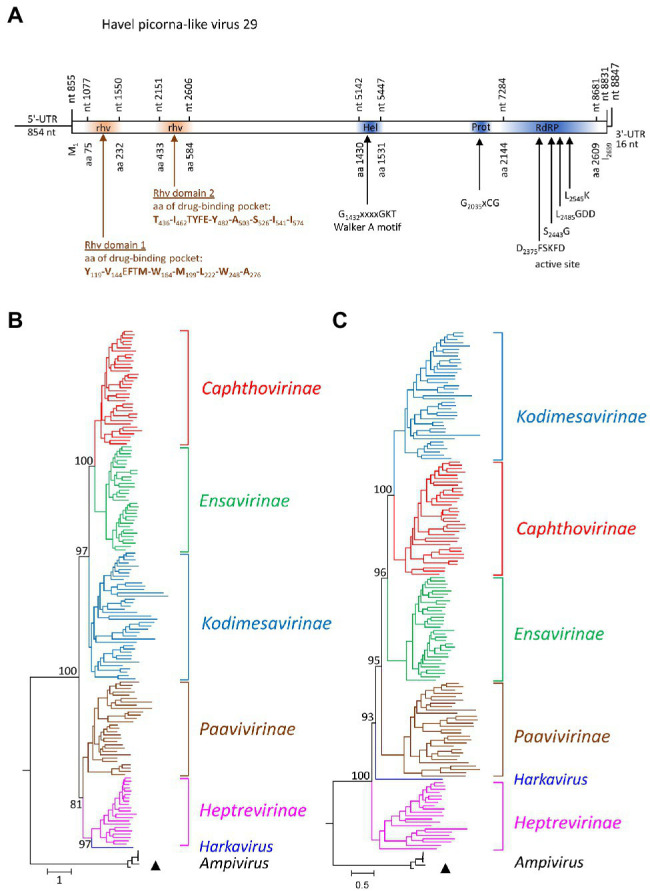
Analysis of Havel picorna-like virus 29. **(A)** The genome organization corresponds to the picornavirus genome. Conserved domains and sequence motifs were identified by searching the NCBI Conserved Domain Database (https://www.ncbi.nlm.nih.gov/Structure/cdd/wrpsb.cgi). Phylogenetic analyses of the capsid protein-encoding gene region **(B)** and the proteinase/polymerase gene region **(C)** of Havel picorna-like virus 29 and 162 picornavirus strains. The tree was inferred with IQ-Tree 2, optimal substitution model: GTR + F + R9 **(B)** and GTR + F + R8 **(C)**. The respective sub-families and genera are given to the right. A triangle (▲) indicates the virus of the present study. Numbers at nodes present bootstrap values obtained after 50,000 ultrafast bootstrap replications. The scale indicates substitutions per site. More details are presented in Supplementary Figures S4A,B. UTR, untranslated region; Hel, helicase; Prot, proteinase; RdRP, polymerase; and rhv, capsid protein with similarity to the rhinovirus capsid protein with jelly roll fold and drug-binding pocket.

One almost complete picornavirus genome, HPLV-29 (8,847 nt), was detected in the Havel river sample. It shows a typical picornavirus genome organization (Figure 4A) and significant sequence homology (>75%) to the genomes of newt ampivirus (KP770140), Shahe picorna-like virus 13 from freshwater arthropods (KX883649; KX883657), and two virus sequences from cloacal swabs of birds (MT138174; MT138399). Divergences of the capsid protein VP1 of 41% in comparison with newt ampivirus and 35.3% in comparisons with Shahe picorna-like virus 13 and the viruses from avian swabs indicate a putative third genotype, ampivirus A3, within the species *Ampivirus A*. Important unique differences are a long insertion of 32 aa in VP1 and an N-terminal deletion of 41 aa of 3A (data not shown). Phylogenetic analyses of the P1- and 3CD-encoding gene regions confirm the close relation of HPLV-29 to ampiviruses (Figures 4B,C; Supplementary Figures 4A,B). In addition to HPLV-29, one short contig corresponding to ampivirus A1 was obtained (data not shown). Further five contigs with similarity to the known ampiviruses suggest the existence of at least two other ampivirus genotypes (data not shown).

### Polycipiviridae

**Figure 5:**
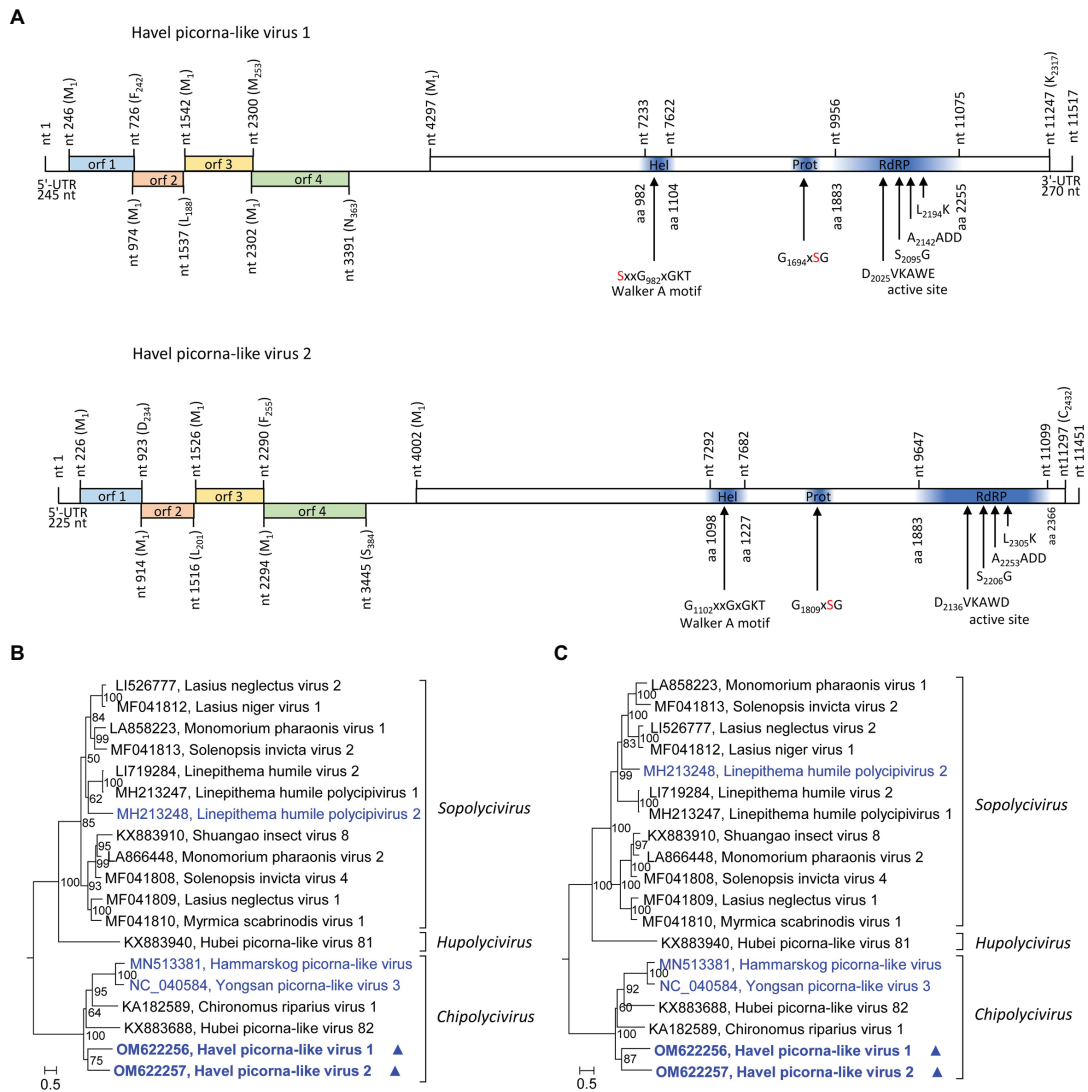
Analysis of Havel picorna-like viruses 1 and 2. **(A)** Genome organization of Havel picorna-like virus 1 (top) and 2 (below). Conserved domains and sequence motifs were identified by searching the NCBI Conserved Domain Database (https://www.ncbi.nlm.nih.gov/Structure/cdd/wrpsb.cgi). Phylogenetic analyses of the concatenated capsid proteins-encoding ORFs 1, 3, and 4 **(B)** and the helicase, proteinase, and polymerase gene regions of ORF5 **(C)**. Sequences of 17 polycipivirus strains plus Havel picorna-like virus 1 and 2 were included. The tree was inferred with IQ-Tree 2; optimal substitution model: GTR + F + R4 in both analyses. Presented are GenBank acc. nos. and virus names. The respective genera are presented to the right. Unassigned viruses are printed in blue. A triangle (▲) indicates the viruses of the present study. Numbers at nodes indicate bootstrap values obtained after 50,000 ultrafast bootstrap replications. The scale indicates substitutions per site. orf, open reading frame; UTR, untranslated region; Hel, helicase; Prot, proteinase; and RdRP, polymerase.

HPLV-1 (11,517 nt) and −2 (11,670 nt) are candidate viruses of the genus *Chipolycivirus*, family *Polycipiviridae*. Their almost complete genomes have five ORFs with ORFs 1, 3, and 4 encoding capsid proteins with jelly roll domains and ORF5 a polyprotein with helicase, proteinase, and polymerase domains. Both viruses have GxSG active site sequences of their proteinase and AADD active site sequences of their RdRP, which is characteristic for chipoliciviruses (Figure 5A). Concatenated sequences of the three CP-encoding ORFs suggest affiliation to the chipolyciviruses (Figure 5B). This result was confirmed with an alignment of ORF5 sequences representing the helicase, proteinase, and polymerase gene regions (Figure 5C). At least 15 short contigs with high similarity to polycipiviruses indicated the occurrence of additional viruses of this family in the Havel river.

### Secoviridae

No viruses with strong similarity to secoviruses were detected in our sample even though DIAMOND suggested several contigs.

### Solinviviridae

**Figure 6:**
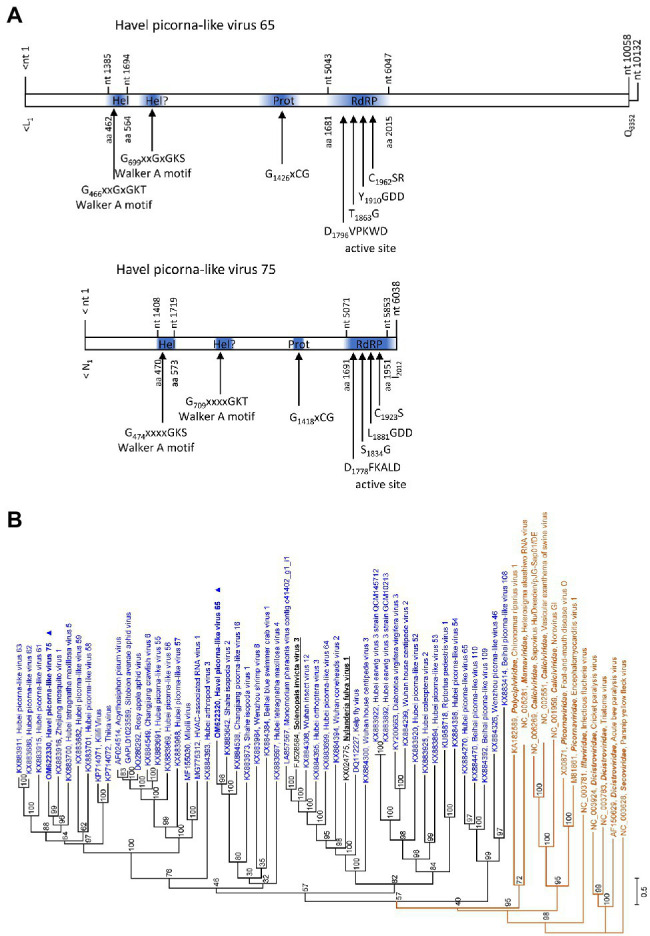
Analysis of solinvivirus-like viruses. **(A)** Genome organization of Havel picorna-like virus 65 (top) and −75 (below). Conserved domains and sequence motifs were identified by searching the NCBI Conserved Domain Database (https://www.ncbi.nlm.nih.gov/Structure/cdd/wrpsb.cgi). **(B)** Phylogenetic analyses of the proteinase/polymerase-encoding gene regions. Sequences of Havel picorna-like virus 65, −75, and two acknowledged solinviviruses (printed in bold and underlined), 47 unassigned solinvivirus candidates, and 12 reference viruses of the order *Picornavirales* (printed in brown) were included. The tree was inferred with IQ-Tree 2; optimal substitution model: TVM + F + R6. Presented are GenBank acc. nos. and virus names as well as genus names for the reference viruses. Unassigned viruses are printed in blue. A triangle (▲) indicates the viruses of the present study. Numbers at nodes indicate bootstrap values obtained after 50,000 ultrafast bootstrap replications. The scale indicates substitutions per site. Hel, helicase; Prot, proteinase; and RdRP, polymerase.

The partial genomes of HPLV-65 (6,945 nt) and HPLV-75 (6,038 nt) exhibit similarity to solinviviruses and encode nonstructural proteins. Most notably, both viruses exhibit a second domain with homology to P-loop ATPases (Figure 6A), a feature which is shared with the two acknowledged solinviviruses and a great number of related, unclassified candidate viruses. The proteinase/polymerase gene region of two solinviviruses (Solenopsis invicta virus 3; Nylanderia fulva virus 1), additional 47 candidate solinviviruses, HPLV-65, and −75 and 12 reference strains of other *Picornavirales* families was investigated in a phylogenetic analysis (Figure 6B). The data revealed a monophyletic branch with solinvivirus-like viruses plus several clades of viruses which despite some genetic diversity are characterized by a second helicase domain with a Walker A motif.

### Viruses With Unusual Genome Organization

**Figure 7:**
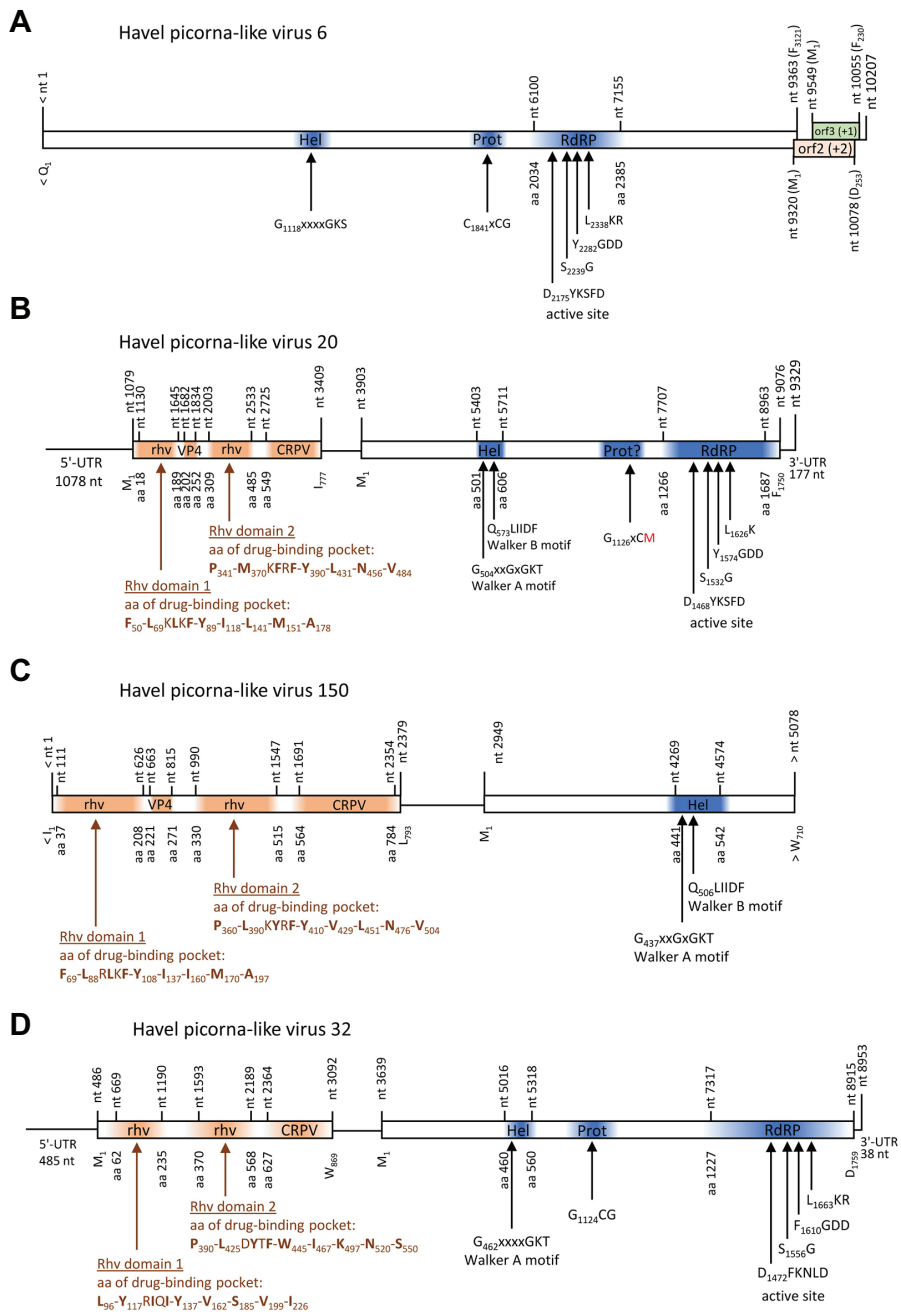
Viruses with unusual genome layout. Genome organization of Havel picorna-like virus 6 **(A)**, −20 **(B)**, −150 **(C),** and −32 **(D)**. Conserved domains and sequence motifs were identified by searching the NCBI Conserved Domain Database (https://www.ncbi.nlm.nih.gov/Structure/cdd/wrpsb.cgi). UTR, untranslated region; Hel, helicase; Prot, proteinase; RdRP, polymerase; rhv, capsid protein with similarity to the rhinovirus capsid protein with jelly roll fold and drug-binding pocket; VP4, viral protein 4; and CRPV, capsid protein with similarity to the cricket paralysis virus capsid protein.

One contig of 10,207 nt exhibited similarity to satsuma dwarf virus (GenBank acc. no. BAA74537; e-value: 7.6e-26) by DIAMOND analysis and to maize chlorotic dwarf virus, potato U virus, and strawberry mottle virus of the *Secoviridae* by BLASTp search. This contig encodes a long ORF with helicase, proteinase, and RdRP domains plus two partly overlapping ORFs in the +1 and + 2 frames at the 3′-end (Figure 7A). No similarities of ORFs 2 and 3 to known proteins were found in additional BLAST and Pfam Conserved Domain Database searches. Phylogenetic analyses using the polymerase and proteinase/polymerase sequences (Supplementary Figure 1), as well as the helicase sequence (data not shown) yielded inconsistent results and failed to confirm a close relationship to secoviruses.

Three contigs, HPLV-32, −20, and −150, belonged to virus genomes with dicistronic layout but with the CP-encoding sequence located 5′ to the NSP-encoding gene region (Figure 7). Whereas almost complete genomes were obtained for HPLV-20 (9,252 nt) and -32 (8,785 nt), only a partial genome of HPLV-150 is available which is comprised of the CP region and the helicase domain of the NSP (5,078 nt). HPLV-32 clustered with other viruses with similar genome organization, namely, Trichosanthes kirilowii picorna-like virus pt111-pic-5, bat badiciviruses 1 and 2, the Aphis glycines virus 1, and soybean-associated bicistronic virus. Surprisingly, HPLV-20 grouped with the kusarnaviruses, indicating independent evolution. Phylogenetic analysis of the helicase indicates close relationship of HPLV-20 and -150 (data not shown).

## Discussion

Next,-generation sequencing techniques provide a powerful tool to analyze even unculturable viruses in various ecosystems. However, there is a disconcerting disparity in the small number of virus isolates with known hosts on the one hand and a plethora of sequences of uncultured viruses obtained from various sources on the other hand. For example, whereas the eight cultured viruses of the *Marnaviridae* infect protists like diatoms, raphidophyte, and thraustochytrids (Lang et al., 2021; Sadeghi et al., 2021),^4^ at least a hundred times more candidate virus sequences are available in GenBank, obtained from very diverse sources such as environmental water and tissue/organ samples of marine invertebrates (e.g., Shi et al., 2016; Vlok et al., 2019; Wolf et al., 2020).

Previous studies of marine water samples have demonstrated a relative dominance of archaeal phages and bacteriophages, giant viruses, and single-stranded DNA and RNA viruses, but there is still a “vast viral unknown” (e.g., Hurwith and Sullivan, 2013; Labonté and Suttle, 2013; Schmidt et al., 2014; Roux et al., 2016; Liang et al., 2019; Callanan et al., 2020). At present, viromes of marine ecosystems are better studied than those of freshwater habitats and soils, even though interest in viruses of lotic and limnic systems, of the phytobiome or soils received increasing interest in recent years (Peduzzi, 2015; Williamson et al., 2017; Schoelz and Stewart, 2018; Roy et al., 2020). Meanwhile, available data indicate a considerably higher abundance of planktonic viruses in lakes than in sea water (Peduzzi, 2015). In the present study, we attempted a contribution to the understanding of virus diversity in a river. For this, we analyzed the enriched virus particles of a 50-liter water sample of the river Havel, taken within the metropolitan area. This river section is characterized by a near-natural river course with both recreational use and seasonal carefully controlled discharge of a wastewater treatment plant as well as occasionally drain water of the city of Berlin after heavy rainfalls. As characteristic for urban areas, the natural base discharge to this river is low, but wastewater effluent contributions under mean minimum discharge conditions vary from 30 to 50% (Karakurt et al., 2019). Previous analyses over a period of several years demonstrated little but significant pollution with human viruses like noroviruses, adenoviruses, hepatitis-E viruses, or cosaviruses, most notably in winter time, due to elaborate water safety management in the summer season (Beyer et al., 2020 and unpublished data and reports by the German Environment Agency). In our metagenomic river virome study, based on a large volume water sample from summer time, no PLVs similar to human, animal, or plant pathogens were detected. Likewise, posaviruses or related husa-, rasa-, basa-, or fisaviruses were also not identified. The latter ones are apparently apathogenic, enteric viruses indicating fecal pollution. This observation can also be explained by the assimilative capacity of the Havel river in summertime at elevated temperatures and high solar UV radiation. However, it should also be kept in mind that there is so far limited knowledge about the efficiency and putative inherent biases associated with virus enrichment protocols on metagenome analyses of viral richness, as demonstrated by Hjelmsø et al. (2017) for sewage metagenome analyses.

A total of 5,687 of 484,430 metaSPAdes scaffolds (1.17%) and 3,902 of 162,082 CLC contigs (2.4%) were identified by DIAMOND as PLV sequences (Taxonomy ID 464095). However, only 41.6 and 41.3%, respectively, of this fraction were assigned to a family, but many family and genus assignments are presumably incorrect as numerous randomly selected contigs were revealed to be misassigned (data not shown). Therefore, we adopted an alternative approach to identify contigs with similarity to one of the 12 *Picornavirales* families/sub-families in a tBLASTx search. Seventy-one almost complete HPLV genomes plus 93 partial genomes with lengths up to 10.95 kb were identified with this approach. At least 72 HPLVs are *Marnaviridae* candidates and about 60 were included in the pol, prot/pol, and CP trees (Figure 1, Supplementary Figures 1, 2). Whereas all so far acknowledged marnaviruses were detected in marine diatoms, unicellular algae, and heterotrophic protists, all recently proposed marnaviruses were from marine animals, marine algae, and coastal or estuarine water samples (Vlok et al., 2019; Lang et al., 2021). Presence of marnavirus candidates in the river Havel is compatible with the hypothesis that protists of freshwater habitats are also competent hosts. The present study, however, provides no data on possible host species. It is surprising that many marnavirus-like PLVs have been detected in plants, fecal samples, or cloacal swabs (e.g., sequences which were used in our phylogenetic analyses: GenBank acc. nos. KX644944, KY926885, MG995720, MN917672, MN917673, MN917674, MN823682, MN823683, MN823684, MN823685, MN823686, MN823687, MN823689, MN823691, MN823692, MT138127, MT138128, MT138129, MT138130, MT138131, MT138132, MT138133, and MT138336). Presence of PLVs in fecal samples or cloacal swabs is often explained by uptake of contaminated food or water. Occurrence of PLVs in plants of terrestrial habitats, however, requires either plant-protist contacts in the phytobiome or virus uptake *via* roots as has been shown for enteric viruses and bacteriophages (Murphy and Syverton, 1958; Ward and Mahler, 1982; Katzenelson and Mills, 1984; Urbanucci et al., 2009; Hirneisen et al., 2012).

Besides HPLV-102, −141, and − 159, no other dicistroviruses of the three genera *Aparavirus*, *Cripavirus*, and *Triatovirus* were identified among our HPLVs (Figure 1, Supplementary Figure 1). However, 50 sequences with significant similarity to dicistroviruses were detected. Three of four virus groups cluster close to sequences of the three dicistrovirus genera with high bootstrap values (>89% in the proteinase/polymerase tree). Other robust clades of the tree contain both viruses with dicistronic and monocistronic genomes. All clades are detectable in the CP tree indicating yet undefined virus groups which may represent new taxa. Of special interest is a clade comprising six dicistronic viruses with a CP polyprotein at the 5′-end of the genome.

HPLV-90, −93, and − 126 are calici-like viruses with similarity to viruses from lizards, bats, an unspecified insect, marine annelids, and bivalve shellfish. These viruses comprise a separate clade in both the calicivirus VP1 tree and the proteinase/polymerase tree (Figures 2B,C). All acknowledged caliciviruses infect vertebrates, either mammals or fish. Detection in insects or bivalves may suggest invertebrate hosts but more likely contamination. Presence of such viruses in Havel river water and in filtrating shellfish is compatible with the assumption of a fish host and virus release into water. However, interspecies infection and ocean reservoirs have been described (Smith et al., 1980, 1998) and fish may be one of several possible hosts.

The *Picornaviridae* family is among the most divergent virus families in the virosphere. Sixty-eight genera, 158 species, and more than 650 types have been described (Zell et al., 2017).^5^ The genome sequence of the first picornavirus infecting a fish was published in 2013 but descriptions of PLVs in fish, amphibia, and reptiles date back in the 1980s (Fichtner et al., 2013 and literature cited therein). Since then, numerous picornaviruses have been detected in lower vertebrates and meanwhile more than one hundred of such viruses are known (Shi et al., 2018). Therefore, it was surprising to detect only one picornavirus in Havel river water. HPLV-29 belongs to a third ampivirus type and additional sequences of the Havel river suggest the existence of further two ampivirus genotypes (Supplementary Figures 4A,B). The ampivirus was originally detected in fecal samples of smooth newts (Reuter et al., 2015). Later, similar viruses were found in unspecified freshwater arthropods (Shi et al., 2016) and (misclassified as totivirus) in cloacal swabs of flamingos and red-crowned cranes (see GenBank acc. nos. MT138174 and MT138399). As picornaviruses infect only vertebrates, freshwater arthropods are unlikely hosts but may have accumulated the virus in their gills. Experimental and natural accumulation of polioviruses and other enteroviruses in marine shellfish has been described (e.g., Hedström and Lycke, 1964; Metcalf and Stiles, 1965). Detection of ampiviruses in the Havel river water is compatible with the assumption of newts and other amphibia as hosts. However, a final answer whether amphibia or birds are true hosts of ampiviruses has to await virus isolation and clinical or experimental data.

Solinviviruses have only recently been described (Valles et al., 2014). We detected two viruses which belong to a monophyletic cluster of viruses comprised of the two acknowledged solinviviruses and a great number of related, unclassified candidate viruses (Brown et al., 2019).^6^ One interesting feature of these viruses is the presence of a second protein domain with similarity to P-loop ATPases. All solinvivirus candidates have a second Walker A motif, either a complete version (GxxGxGK^S^/_T_) or a modified one.

Two viruses with unusual genome layouts, HPLV-20 and -150, exhibit marked similarity to members of the *Marnaviridae* family. Whereas the CP polyprotein shows similarity to the capsid of sogarnaviruses, proteinase and polymerase are kusarnavirus-like (Supplementary Figures 1, 2). Also the helicase is kusarnavirus-like (data not shown). Similar observations were described by Vlok et al. (2019). Several genera of the *Marnaviridae* exhibit a striking feature: mono- and dicistronic viruses are found in the same genus which is unusual in virus taxonomy. It has to be awaited additional sequence data to decide whether the genome layout of HPLV-20 is an exceptional feature or a third theme of marnavirus genome organization. Further, it is likely that some of our HPLVs represent members of novel virus genera and/or families. (i) Among such viruses are HPLV-9, a micalovirus-like dicistronic virus which clusters distinct from the known members of the *Picornavirales* but also distinct from other PLVs (see Figure 1, Supplementary Figure S1). Further candidates of novel families are the HPLVs with unusual genome layouts. (ii) The genome of HPLV-6 presents 3 ORFs with a missing 5′-end of ORF1 (Figure 7). Even so, ORF1 encodes a polyprotein of at least 3,121 aa which is rather long. No capsid proteins with jelly rolls motifs were detected by the Pfam conserved domain search tool. Also ORFs 2 and 3 lack similarity to other proteins of the NCBI protein database. However, presence of a Walker A motif, a CxCG proteinase active site sequence motif, and the characteristic RdRP active site motifs suggest a PLV. (iii) Another virus of interest is HPLV-32, a dicistronic virus with CP-encoding ORFs at the 5′-end of the genome (Figure 7). This virus clusters with Trichosanthes kirilowii picorna-like virus strain pt111-pic-5 and four other viruses with similar genome layout. Two of these viruses are from soybean aphids and two from feces of the straw-colored fruit bat (Yinda et al., 2017). Neither *Trichosanthes*, a plant of the cucumber family, nor aphids or bats are the likely hosts, but may be contaminated or may have ingested virus by food or water intake. As part of a complex phytobiome, aphids or fruit bats may play a role in virus distribution as well as the Havel river. (iii) Of note, viruses with monocistronic genome layouts but similarity of their proteinase/polymerase proteins to dicistroviruses are good candidates for novel virus taxa, as *per definitionem* they cannot be “dicistroviruses.”

## Conclusion

The Havel river in Berlin, Germany, is a near-natural river. It harbors highly divergent PLVs, most of which belong to the *Marnaviridae* family or are distant relatives of dicistroviruses likely representing new virus taxa. Whereas the majority of marnaviruses has been detected in seawater previously, an increasing number of marnavirus candidates are from freshwater habitats or terrestrial organisms. Dicistroviruses have been isolated from various arthropods, but there are many dicistrovirus-like sequences obtained from environmental water samples, plants, and vertebrate hosts. Both examples indicate more complex ecological connections of PLVs and their hosts. The role of rivers and lakes in the transmission and distribution of PLVs is so far insufficiently investigated and deserves more efforts to explore its relevance. However, as long as hosts are unknown, transmission cycles and the ecological importance of PLVs in the environment will remain obscure. Continuation of sequence data collection and progress in taxonomic classification are indispensable for an appropriate and advanced description of the virosphere diversity.

## Data Availability Statement

The datasets presented in this study can be found in online repositories. The names of the repository/repositories and accession number(s) can be found at: NCBI BioProject – PRJNA803428, BioSample SAMN25651207, GenBank (OM622256-OM622421).

## Author Contributions

RZ and H-CS: conception and study design and manuscript preparation. H-CS: responsibility for sampling, transport, and large scale virus enrichments. RZ: RNA preparation. MG: sequencing and sequence data processing. MG, RZ, and LS: data curation, bioinformatic analysis, and phylogenetic analyses. All authors have read and approved the final version of the manuscript.

## Conflict of Interest

The authors declare that the research was conducted in the absence of any commercial or financial relationships that could be construed as a potential conflict of interest.

## Publisher’s Note

All claims expressed in this article are solely those of the authors and do not necessarily represent those of their affiliated organizations, or those of the publisher, the editors and the reviewers. Any product that may be evaluated in this article, or claim that may be made by its manufacturer, is not guaranteed or endorsed by the publisher.

## Footnotes

^1^ https://talk.ictvonline.org/taxonomy/

^2^ https://ftp.ncbi.nlm.nih.gov/blast/executables/blast+/2.6.0

^3^ https://www.ncbi.nlm.nih.gov/Structure/cdd/wrpsb.cgi

^4^ www.ictv.global/report/marnaviridae

^5^ www.ictv.global/report/picornaviridae

^6^ www.ictv.global/report/solinviviridae

## Supplementary Material

Supplementary Figure 1 Phylogenetic analysis of polymerase (left) and proteinase/polymerase-encoding sequences of dicistroviruses and marnaviruses. Two-hundred sixty-seven sequences of acknowledged dicistroviruses, marnaviruses and unassigned candidate viruses were aligned with MEGA for the polymerase tree, 264 sequences for the proteinase/polymerase tree. The trees were inferred with IQ-Tree 2, optimal substitution model: GTR + F + R9 for the pol tree and GTR + F + R10 for the prot/pol tree, respectively. Numbers at nodes present bootstrap values obtained after 50,000 ultrafast bootstrap replications. The scale indicates substitutions per site. Presented are GenBank acc. nos. and virus names. The respective genera are indicated. Colour code: aparaviruses, dark brown; bacillarnaviruses, blue; cripaviruses, light blue; kusarnaviruses, ochre; labyrnaviruses, light green; locarnaviruses, dark blue; marnaviruses, magenta; salisharnaviruses, dark green; sogarnaviruses, red; triatoviruses, brown, untypeable viruses, black. A triangle (▲) indicates viruses of the present study. Blue boxes indicate viruses with unusual genome layout (capsid protein-encoding gene region at 5'-end, nonstructural polyprotein-encoding gene region at the 3′-end). Yellow boxes indicate four dicistrovirus-like sequence clusters.

Supplementary Figure 2 Phylogenetic analysis of the capsid protein-encoding sequences of dicistroviruses and marnaviruses. Two-hundred forty-two sequences of acknowledged dicistroviruses, marnaviruses and unassigned candidate viruses were aligned with MEGA. The tree was inferred with IQ-Tree 2, optimal substitution model: GTR + F + R9. Numbers at nodes present bootstrap values obtained after 50,000 ultrafast bootstrap replications. The scale indicates substitutions per site. Presented are GenBank acc. nos. and virus names. The respective genera are indicated. Colour code: aparaviruses, dark brown; bacillarnaviruses, blue; cripaviruses, light blue; kusarnaviruses, ochre; labyrnaviruses, light green; locarnaviruses, dark blue; marnaviruses, magenta; salisharnaviruses, dark green; sogarnaviruses, red; triatoviruses, brown, untypeable viruses, black. A triangle (▲) indicates viruses of the present study.

Supplementary Figure 3 Phylogenetic analysis of the VP1-to-helicase gene region of 15 acknowleged iflavirus strains, 20 candidate strains and Havel picorna-like viruses 14 and -129. The tree was inferred with IQ-Tree 2, optimal substitution model: GTR + F + R5. Numbers at nodes present bootstrap values obtained after 50,000 ultrafast bootstrap replications. The scale indicates substitutions per site. Presented are GenBank acc. nos. and virus names. Unassigned viruses are printed in blue. A triangle (▲) indicates the viruses of the present study.

Supplementary Figure 4 **(A)** Phylogenetic analysis of the capsid protein-encoding gene region (P1) of Havel picorna-like virus 29 and 162 picornaviruses. The tree was inferred with IQ-Tree 2, optimal substitution model: GTR+F+R9. Presented are GenBank acc. nos., genera (printed in italics and bold), virus names and strain designations (in square brackets). Sub-family names where available are given to the right. A triangle (▲) indicates the virus of the present study. Numbers at nodes present bootstrap values obtained after 50,000 ultrafast bootstrap replications. The scale indicates substitutions per site. **(B)** Phylogenetic analysis of the proteinase/polymerase-encoding gene region (3CD) of Havel picorna-like virus 29 and 162 picornaviruses. The tree was inferred with IQ-Tree 2, optimal substitution model: GTR+F+R8. Presented are GenBank acc. nos., genera (printed in italics and bold), virus names and strain designations (in square brackets). Sub-family names where available are given to the right. A triangle (▲) indicates the virus of the present study. Numbers at nodes present bootstrap values obtained after 50,000 ultrafast bootstrap replications. The scale indicates substitutions per site.
