# Supplementary material for: Picorna-Like Viruses of the Havel River, Germany
Source: Front Microbiol. 2022 Apr 4;13:865287. doi: 10.3389/fmicb.2022.865287 (PMC9013969; doi:10.3389/fmicb.2022.865287)
Supplement: Supplementary Figure 1 — Phylogenetic analysis of polymerase (left) and proteinase/polymerase-encoding sequences of dicistroviruses and marnaviruses. Two-hundred sixty-seven sequences of acknowledged dicistroviruses, marnaviruses and unassigned candidate viruses were aligned with MEGA for the polymerase tree, 264 sequences for the proteinase/polymerase tree. The trees were inferred with IQ-Tree 2, optimal substitution model: GTR + F + R9 for the pol tree and GTR + F + R10 for the prot/pol tree, respectively. Numbers at nodes present bootstrap values obtained after 50,000 ultrafast bootstrap replications. The scale indicates substitutions per site. Presented are GenBank acc. nos. and virus names. The respective genera are indicated. Colour code: aparaviruses, dark brown; bacillarnaviruses, blue; cripaviruses, light blue; kusarnaviruses, ochre; labyrnaviruses, light green; locarnaviruses, dark blue; marnaviruses, magenta; salisharnaviruses, dark green; sogarnaviruses, red; triatoviruses, brown, untypeable viruses, black. A triangle (▲) indicates viruses of the present study. Blue boxes indicate viruses with unusual genome layout (capsid protein-encoding gene region at 5'-end, nonstructural polyprotein-encoding gene region at the 3′-end). Yellow boxes indicate four dicistrovirus-like sequence clusters. [file Data_Sheet_1.PDF]

Virus	GenBank acc. No.	Comments	Length (nt)	Preliminary assignment (family/genus)
Havel picorna-like virus 1	OM622256	almost complete genome	11517	*Polycipiviridae* /*Sopolycivirus*
Havel picorna-like virus 2	OM622257	almost complete genome	11670	*Polycipiviridae* /*Sopolycivirus*
Havel picorna-like virus 3	OM622258	almost complete genome	11398	untypeable
Havel picorna-like virus 4	OM622259	almost complete genome	10948	untypeable
Havel picorna-like virus 5	OM622260	almost complete genome	11229	untypeable
Havel picorna-like virus 6	OM622261	partial genome	10207	untypeable
Havel picorna-like virus 7	OM622262	almost complete genome	10121	Dicistrovirus-like cluster 1
Havel picorna-like virus 8	OM622263	almost complete genome	9957	untypeable
Havel picorna-like virus 9	OM622264	partial genome	9903	untypeable
Havel picorna-like virus 10	OM622265	almost complete genome	9807	Dicistrovirus-like cluster 3
Havel picorna-like virus 11	OM622266	almost complete genome	9803	Dicistrovirus-like cluster 1
Havel picorna-like virus 12	OM622267	almost complete genome	9642	*Marnaviridae* /*Labyrnavirus*
Havel picorna-like virus 13	OM622268	almost complete genome	9539	*Marnaviridae* /*Labyrnavirus*
Havel picorna-like virus 14	OM622269	almost complete genome	9465	*Iflaviridae*
Havel picorna-like virus 15	OM622270	almost complete genome	9412	untypeable
Havel picorna-like virus 16	OM622271	almost complete genome	9413	untypeable
Havel picorna-like virus 17	OM622272	almost complete genome	9408	untypeable
Havel picorna-like virus 18	OM622273	almost complete genome	9333	untypeable
Havel picorna-like virus 19	OM622274	almost complete genome	9278	*Marnaviridae* /*Salisharnavirus*
Havel picorna-like virus 20	OM622275	almost complete genome	9329	*Marnaviridae* /untypeable
Havel picorna-like virus 21	OM622276	almost complete genome	9503	Dicistrovirus-like cluster 2
Havel picorna-like virus 22	OM622277	almost complete genome	9256	*Marnaviridae* /*Locarnavirus*
Havel picorna-like virus 23	OM622278	almost complete genome	9205	Dicistrovirus-like cluster 2
Havel picorna-like virus 24	OM622279	almost complete genome	9176	*Marnaviridae* /*Locarnavirus*
Havel picorna-like virus 25	OM622280	almost complete genome	9243	untypeable
Havel picorna-like virus 26	OM622281	almost complete genome	9049	*Marnaviridae* /*Locarnavirus*
Havel picorna-like virus 27	OM622282	almost complete genome	8986	*Marnaviridae* /*Locarnavirus*
Havel picorna-like virus 28	OM622283	almost complete genome	8854	*Marnaviridae* /*Locarnavirus*
Havel picorna-like virus 29	OM622284	almost complete genome	8847	*Picornaviridae* /*Ampivirus*
Havel picorna-like virus 30	OM622285	partial genome	2182	untypeable
Havel picorna-like virus 31	OM622286	almost complete genome	8810	*Marnaviridae* /*Locarnavirus*
Havel picorna-like virus 32	OM622287	almost complete genome	8953	untypeable
Havel picorna-like virus 33	OM622288	almost complete genome	8776	untypeable
Havel picorna-like virus 34	OM622289	almost complete genome	8813	Dicistrovirus-like cluster 3
Havel picorna-like virus 35	OM622290	almost complete genome	8998	Dicistrovirus-like cluster 3
Havel picorna-like virus 36	OM622291	almost complete genome	8730	Dicistrovirus-like cluster 3
Havel picorna-like virus 37	OM622292	almost complete genome	8600	*Marnaviridae* /*Locarnavirus*
Havel picorna-like virus 38	OM622293	almost complete genome	9428	Marnaviridae/untypeable
Havel picorna-like virus 39	OM622294	almost complete genome	8867	*Marnaviridae* /*Locarnavirus*
Havel picorna-like virus 40	OM622295	almost complete genome	8701	Marnaviridae/untypeable
Havel picorna-like virus 41	OM622296	almost complete genome	8646	*Marnaviridae* /*Locarnavirus*
Havel picorna-like virus 42	OM622297	almost complete genome	9021	*Marnaviridae* /*Locarnavirus*
Havel picorna-like virus 43	OM622298	almost complete genome	8567	Marnaviridae/untypeable
Havel picorna-like virus 44	OM622299	almost complete genome	8546	*Marnaviridae* /*Locarnavirus*
Havel picorna-like virus 45	OM622300	almost complete genome	8484	*Marnaviridae* /*Locarnavirus*
Havel picorna-like virus 46	OM622301	almost complete genome	8506	Dicistrovirus-like cluster 3
Havel picorna-like virus 47	OM622302	almost complete genome	8368	Dicistrovirus-like cluster 3
Havel picorna-like virus 48	OM622303	almost complete genome	8315	*Marnaviridae* /*Locarnavirus*
Havel picorna-like virus 49	OM622304	almost complete genome	8282	Dicistrovirus-like cluster 4
Havel picorna-like virus 50	OM622305	almost complete genome	8004	Dicistrovirus-like cluster 4
Havel picorna-like virus 51	OM622306	almost complete genome	7963	Dicistrovirus-like cluster 4
Havel picorna-like virus 52	OM622307	almost complete genome	8831	*Marnaviridae* /untypeable
Havel picorna-like virus 53	OM622308	almost complete genome	8980	Dicistrovirus-like cluster 3
Havel picorna-like virus 54	OM622309	almost complete genome	8014	Dicistrovirus-like cluster 4
Havel picorna-like virus 55	OM622310	almost complete genome	7887	Dicistrovirus-like cluster 4
Havel picorna-like virus 56	OM622311	partial genome	7858	Dicistrovirus-like cluster 3
Havel picorna-like virus 57	OM622312	partial genome	8059	Dicistrovirus-like cluster 1
Havel picorna-like virus 58	OM622313	almost complete genome	7786	Dicistrovirus-like cluster 4
Havel picorna-like virus 59	OM622314	almost complete genome	7751	Dicistrovirus-like cluster 4
Havel picorna-like virus 60	OM622315	partial genome	8173	untypeable
Havel picorna-like virus 61	OM622316	partial genome	9045	*Marnaviridae* /*Sogarnavirus*
Havel picorna-like virus 62	OM622317	almost complete genome	8508	Dicistrovirus-like cluster 3
Havel picorna-like virus 63	OM622318	almost complete genome	7931	Dicistrovirus-like cluster 3
Havel picorna-like virus 64	OM622319	almost complete genome	8976	*Marnaviridae* /*Sogarnavirus*
Havel picorna-like virus 65	OM622320	partial genome	10130	*Solinviviridae*
Havel picorna-like virus 66	OM622321	almost complete genome	9244	untypeable
Havel picorna-like virus 67	OM622322	partial genome	6730	*Marnaviridae* /*Labyrnavirus*
Havel picorna-like virus 68	OM622323	partial genome	6638	*Marnaviridae* /*Labyrnavirus*
Havel picorna-like virus 69	OM622324	partial genome	6627	*Marnaviridae* /*Locarnavirus*
Havel picorna-like virus 70	OM622325	partial genome	6544	*Marnaviridae* /*Labyrnavirus*
Havel picorna-like virus 71	OM622326	partial genome	6336	*Marnaviridae* /*Locarnavirus*
Havel picorna-like virus 72	OM622327	partial genome	6286	*Marnaviridae* /*Salisharnavirus*
Havel picorna-like virus 73	OM622328	partial genome	6056	Dicistrovirus-like cluster 3
Havel picorna-like virus 74	OM622329	partial genome	6038	untypeable
Havel picorna-like virus 75	OM622330	partial genome	6037	*Solinviviridae*
Havel picorna-like virus 76	OM622331	almost complete genome	10012	Dicistrovirus-like cluster 1
Havel picorna-like virus 77	OM622332	almost complete genome	9157	*Marnaviridae* /*Salisharnavirus*
Havel picorna-like virus 78	OM622333	almost complete genome	8611	*Marnaviridae* /*Locarnavirus*
Havel picorna-like virus 79	OM622334	partial genome	2085	*Marnaviridae* /*Locarnavirus*
Havel picorna-like virus 80	OM622335	almost complete genome	7897	Dicistrovirus-like cluster 4
Havel picorna-like virus 81	OM622336	partial genome	7995	untypeable
Havel picorna-like virus 82	OM622337	almost complete genome	7634	Dicistrovirus-like cluster 4
Havel picorna-like virus 83	OM622338	partial genome	9797	Dicistrovirus-like cluster 1

Havel picorna-like virus 84	OM622339	almost complete genome	9057	*Marnaviridae* / *Locarnavirus*
Havel picorna-like virus 85	OM622340	almost complete genome	8930	*Marnaviridae* / *Locarnavirus*
Havel picorna-like virus 86	OM622341	partial genome	5244	untypeable
Havel picorna-like virus 87	OM622342	almost complete genome	8910	Dicistrovirus-like cluster 4
Havel picorna-like virus 88	OM622343	partial genome	4884	Dicistrovirus-like cluster 4
Havel picorna-like virus 89	OM622344	partial genome	5400	*Marnaviridae* / *Locarnavirus*
Havel picorna-like virus 90	OM622345	partial genome	2746	*Caliciviridae*
Havel picorna-like virus 91	OM622346	partial genome	5453	*Marnaviridae* / *Salisharnavirus*
Havel picorna-like virus 92	OM622347	partial genome	4695	Dicistrovirus-like cluster 2
Havel picorna-like virus 93	OM622348	partial genome	7112	*Caliciviridae*
Havel picorna-like virus 94	OM622349	partial genome	5733	*Marnaviridae* / *Salisharnavirus*
Havel picorna-like virus 95	OM622350	partial genome	5181	Dicistrovirus-like cluster 4
Havel picorna-like virus 96	OM622351	almost complete genome	9179	Dicistrovirus-like cluster 3
Havel picorna-like virus 97	OM622352	partial genome	4381	untypeable
Havel picorna-like virus 98	OM622353	partial genome	4862	Dicistrovirus-like cluster 4
Havel picorna-like virus 99	OM622354	almost complete genome	8909	Dicistrovirus-like cluster 3
Havel picorna-like virus 100	OM622355	partial genome	4264	*Marnaviridae* / *Labyrnavirus*
Havel picorna-like virus 101	OM622356	partial genome	6731	Dicistrovirus-like cluster 3
Havel picorna-like virus 102	OM622357	partial genome	3028	*Dicistroviridae* / *Cripavirus*
Havel picorna-like virus 103	OM622358	partial genome	4175	untypeable
Havel picorna-like virus 104	OM622359	partial genome	2753	*Marnaviridae* / untypeable
Havel picorna-like virus 105	OM622360	partial genome	5432	Dicistrovirus-like cluster 4
Havel picorna-like virus 106	OM622361	partial genome	8187	*Marnaviridae* / *Locarnavirus*
Havel picorna-like virus 107	OM622362	partial genome	3885	Dicistrovirus-like cluster 3
Havel picorna-like virus 108	OM622363	partial genome	3817	*Marnaviridae* / *Kusarnavirus*
Havel picorna-like virus 109	OM622364	partial genome	2502	untypeable
Havel picorna-like virus 110	OM622365	partial genome	3707	Dicistrovirus-like cluster 3
Havel picorna-like virus 111	OM622366	almost complete genome	10826	untypeable
Havel picorna-like virus 112	OM622367	partial genome	3656	untypeable
Havel picorna-like virus 113	OM622368	partial genome	3575	Dicistrovirus-like cluster 4
Havel picorna-like virus 114	OM622369	partial genome	3542	untypeable
Havel picorna-like virus 115	OM622370	partial genome	6075	*Marnaviridae* / *Kusarnavirus*
Havel picorna-like virus 116	OM622371	partial genome	3486	untypeable
Havel picorna-like virus 117	OM633372	partial genome	2446	*Marnaviridae* / untypeable
Havel picorna-like virus 118	OM622373	partial genome	3729	untypeable
Havel picorna-like virus 119	OM622374	partial genome	3904	*Marnaviridae* / *Kusarnavirus*
Havel picorna-like virus 120	OM622375	partial genome	4950	*Marnaviridae* / *Locarnavirus*
Havel picorna-like virus 121	OM622376	partial genome	3243	*Marnaviridae* / *Locarnavirus*
Havel picorna-like virus 122	OM622377	partial genome	3184	*Marnaviridae* / *Labyrnavirus*
Havel picorna-like virus 123	OM622378	partial genome	3394	*Marnaviridae* / *Locarnavirus*
Havel picorna-like virus 124	OM622379	partial genome	3147	Dicistrovirus-like cluster 4
Havel picorna-like virus 125	OM622380	partial genome	3104	*Marnaviridae* / *Locarnavirus*
Havel picorna-like virus 126	OM622381	partial genome	1038	*Caliciviridae*
Havel picorna-like virus 127	OM622382	partial genome	3028	untypeable
Havel picorna-like virus 128	OM622383	partial genome	3682	*Marnaviridae* / *Locarnavirus*
Havel picorna-like virus 129	OM622384	partial genome	3670	*Iflaviridae* / untypeable
Havel picorna-like virus 130	OM622385	partial genome	3127	Dicistrovirus-like cluster 4
Havel picorna-like virus 131	OM622386	partial genome	3819	*Marnaviridae* / *Locarnavirus*
Havel picorna-like virus 132	OM622387	partial genome	2882	Dicistrovirus-like cluster 4
Havel picorna-like virus 133	OM622388	partial genome	1931	*Marnaviridae* / *Locarnavirus*
Havel picorna-like virus 134	OM622389	partial genome	2777	*Marnaviridae* / *Salisharnavirus*
Havel picorna-like virus 135	OM622390	partial genome	2771	untypeable
Havel picorna-like virus 136	OM622391	partial genome	2491	untypeable
Havel picorna-like virus 137	OM622392	partial genome	5998	*Marnaviridae* / *Locarnavirus*
Havel picorna-like virus 138	OM622393	partial genome	2742	*Marnaviridae* / *Sogarnavirus*
Havel picorna-like virus 139	OM622394	partial genome	2715	*Marnaviridae* / *Labyrnavirus*
Havel picorna-like virus 140	OM622395	partial genome	2679	*Marnaviridae* / *Sogarnavirus*
Havel picorna-like virus 141	OM622396	partial genome	3635	*Dicistroviridae* / *Cripavirus*
Havel picorna-like virus 142	OM622397	partial genome	2714	*Marnaviridae* / *Salisharnavirus*
Havel picorna-like virus 143	OM622398	partial genome	2424	*Marnaviridae* / *Labyrnavirus*
Havel picorna-like virus 144	OM622399	partial genome	2632	*Marnaviridae* / *Locarnavirus*
Havel picorna-like virus 145	OM622400	almost complete genome	8940	Dicistrovirus-like cluster 3
Havel picorna-like virus 146	OM622401	partial genome	2564	*Marnaviridae* / *Salisharnavirus*
Havel picorna-like virus 147	OM622402	partial genome	2884	*Marnaviridae* / *Salisharnavirus*
Havel picorna-like virus 148	OM622403	partial genome	2991	*Solinviviridae*
Havel picorna-like virus 149	OM622404	partial genome	3115	Dicistrovirus-like cluster 3
Havel picorna-like virus 150	OM622405	partial genome	5076	*Marnaviridae* / *Sogarnavirus*
Havel picorna-like virus 151	OM622406	partial genome	3922	*Marnaviridae* / *Sogarnavirus*
Havel picorna-like virus 152	OM622407	partial genome	2484	*Marnaviridae* / *Locarnavirus*
Havel picorna-like virus 153	OM622408	partial genome	4856	*Marnaviridae* / *Locarnavirus*
Havel picorna-like virus 154	OM622409	almost complete genome	9003	*Marnaviridae* / *Locarnavirus*
Havel picorna-like virus 155	OM622410	partial genome	2089	untypeable
Havel picorna-like virus 156	OM622411	partial genome	2162	*Marnaviridae* / *Salisharnavirus*
Havel picorna-like virus 157	OM622412	partial genome	2332	untypeable
Havel picorna-like virus 158	OM622413	partial genome	2545	untypeable
Havel picorna-like virus 159	OM622414	partial genome	2316	*Dicistroviridae* / *Cripavirus*
Havel picorna-like virus 160	OM622415	partial genome	2925	Dicistrovirus-like cluster 3
Havel picorna-like virus 161	OM622416	partial genome	2076	untypeable
Havel picorna-like virus 162	OM622417	partial genome	4082	Dicistrovirus-like cluster 3
Havel picorna-like virus 163	OM622418	partial genome	3540	*Marnaviridae* / *Locarnavirus*
Havel picorna-like virus 164	OM622419	partial genome	1134	*Picornaviridae* / *Ampivirus*
Havel picorna-like virus 165	OM622420	partial genome	3028	*Iflaviridae*
Havel picorna-like virus 166	OM622421	partial genome	1330	*Dicistroviridae* / *Cripavirus*
