# Supplementary material for: Picorna-Like Viruses of the Havel River, Germany
Source: Front Microbiol. 2022 Apr 4;13:865287. doi: 10.3389/fmicb.2022.865287 (PMC9013969; doi:10.3389/fmicb.2022.865287)
Supplement: Supplementary Figure 2 — Phylogenetic analysis of the capsid protein-encoding sequences of dicistroviruses and marnaviruses. Two-hundred forty-two sequences of acknowledged dicistroviruses, marnaviruses and unassigned candidate viruses were aligned with MEGA. The tree was inferred with IQ-Tree 2, optimal substitution model: GTR + F + R9. Numbers at nodes present bootstrap values obtained after 50,000 ultrafast bootstrap replications. The scale indicates substitutions per site. Presented are GenBank acc. nos. and virus names. The respective genera are indicated. Colour code: aparaviruses, dark brown; bacillarnaviruses, blue; cripaviruses, light blue; kusarnaviruses, ochre; labyrnaviruses, light green; locarnaviruses, dark blue; marnaviruses, magenta; salisharnaviruses, dark green; sogarnaviruses, red; triatoviruses, brown, untypeable viruses, black. A triangle (▲) indicates viruses of the present study. [file Data_Sheet_2.PDF]

Supplementary Table 2: Estimates of Evolutionary Divergence between VP1 Sequences

[1] Havel picorna-like virus 93
[2] MT341481 Clinch calicivirus 1 isolate CCalV1/C47/2018
[3] MH259583 Bat calicivirus A10 isolate BtCalV/A10/USA/2009
[4] KX884269 Hubei picorna-like virus 77
[5] KM254170 **Bavovirus** Chicken calicivirus isolate CaliciD62/2013
[6] HQ010042 **Bavovirus** Calicivirus chicken/V0021/Bayern/2004
[7] M67473 **Lagovirus** Rabbit hemorrhagic disease virus-FRG
[8] Z69620 **Lagovirus** European brown hare syndrome virus
[9] KX371097 **Minovirus** Fathead minnow calicivirus
[10] JQ347522 **Nacovirus** Turkey calicivirus isolate L11043
[11] KJ473715 **Nacovirus** Goose calicivirus strain N
[12] AY082891 **Nebovirus** Calicivirus strain NB
[13] KT119483 **Nebovirus** Bovine calicivirus strain Kirklareli
[14] M87661 **Norovirus** Norwalk virus
[15] AJ011099 **Norovirus** Bovine calicivirus strain Jena
[16] EU391643 **Recovirus** Tulane virus
[17] JX627575 **Recovirus** WUHARV Calicivirus 1
[18] KJ577140 **Salovirus** Atlantic salmon calicivirus isolate AL V901
[19] KJ577139 **Salovirus** Atlantic salmon calicivirus isolate Nordland/2011
[20] FJ387164 **Sapovirus** pig/sav1/2008/CHN
[21] X86560 **Sapovirus** Sapporo virus-Manchester
[22] AY646856 **Sapovirus** NongKhai-24/Thailand
[23] FJ355928 **Valovirus** Calicivirus pig/AB90/CAN
[24] U76874 **Vesivirus** Vesicular exanthema of swine virus strain A48
[25] M86379 **Vesivirus** Feline calicivirus

[	1	2	3	4	5	6	7	8	9	10	11	12	13	14	15	16	17	18	19	20	21	22	23	24	25]
[1]	-																								
[2]	0.572	-																							
[3]	0.605	0.173	-																						
[4]	0.747	0.741	0.748	-																					
[5]	0.883	0.862	0.875	0.886	-																				
[6]	0.882	0.858	0.873	0.884	0.052	-																			
[7]	0.851	0.837	0.844	0.863	0.794	0.795	-																		
[8]	0.847	0.841	0.855	0.873	0.794	0.792	0.236	-																	
[9]	0.823	0.832	0.834	0.875	0.858	0.859	0.834	0.848	-																
[10]	0.862	0.842	0.842	0.865	0.762	0.766	0.757	0.782	0.867	-															
[11]	0.897	0.874	0.873	0.875	0.764	0.763	0.801	0.798	0.889	0.620	-														
[12]	0.884	0.861	0.877	0.868	0.801	0.800	0.718	0.726	0.870	0.778	0.814	-													
[13]	0.887	0.864	0.859	0.863	0.779	0.774	0.736	0.740	0.864	0.775	0.806	0.578	-												
[14]	0.871	0.881	0.874	0.865	0.848	0.851	0.817	0.828	0.817	0.820	0.835	0.816	0.820	-											
[15]	0.877	0.861	0.871	0.860	0.848	0.852	0.814	0.840	0.841	0.824	0.836	0.799	0.808	0.477	-										
[16]	0.853	0.842	0.843	0.841	0.862	0.859	0.818	0.822	0.814	0.831	0.845	0.848	0.846	0.703	0.698	-									
[17]	0.854	0.841	0.848	0.856	0.849	0.846	0.829	0.831	0.821	0.830	0.856	0.828	0.828	0.711	0.698	0.226	-								
[18]	0.855	0.841	0.840	0.872	0.866	0.862	0.857	0.869	0.741	0.876	0.865	0.860	0.832	0.817	0.834	0.838	0.845	-							
[19]	0.857	0.841	0.846	0.870	0.864	0.860	0.852	0.868	0.734	0.880	0.861	0.851	0.834	0.822	0.838	0.835	0.838	0.171	-						
[20]	0.873	0.844	0.845	0.872	0.755	0.751	0.767	0.772	0.847	0.761	0.752	0.756	0.745	0.829	0.809	0.833	0.824	0.841	0.839	-					
[21]	0.876	0.862	0.871	0.878	0.747	0.748	0.785	0.782	0.879	0.751	0.767	0.764	0.778	0.811	0.829	0.823	0.822	0.855	0.862	0.582	-				
[22]	0.895	0.870	0.871	0.874	0.750	0.747	0.791	0.793	0.886	0.753	0.776	0.758	0.776	0.834	0.842	0.860	0.851	0.867	0.865	0.544	0.477	-			
[23]	0.884	0.861	0.871	0.881	0.848	0.850	0.842	0.850	0.855	0.842	0.847	0.831	0.815	0.723	0.706	0.691	0.676	0.835	0.844	0.805	0.845	0.851	-		
[24]	0.871	0.858	0.861	0.862	0.785	0.790	0.759	0.759	0.878	0.786	0.770	0.760	0.763	0.832	0.830	0.841	0.854	0.856	0.856	0.749	0.727	0.733	0.857	-	
[25]	0.864	0.851	0.860	0.868	0.783	0.781	0.762	0.765	0.850	0.780	0.785	0.762	0.769	0.823	0.820	0.838	0.840	0.867	0.877	0.722	0.709	0.736	0.855	0.463	-
