# Supplementary figures and images for: Picorna-Like Viruses of the Havel River, Germany
Source: Front Microbiol. 2022 Apr 4;13:865287. doi: 10.3389/fmicb.2022.865287 (PMC9013969; doi:10.3389/fmicb.2022.865287)

# Pol

# Prot-Pol

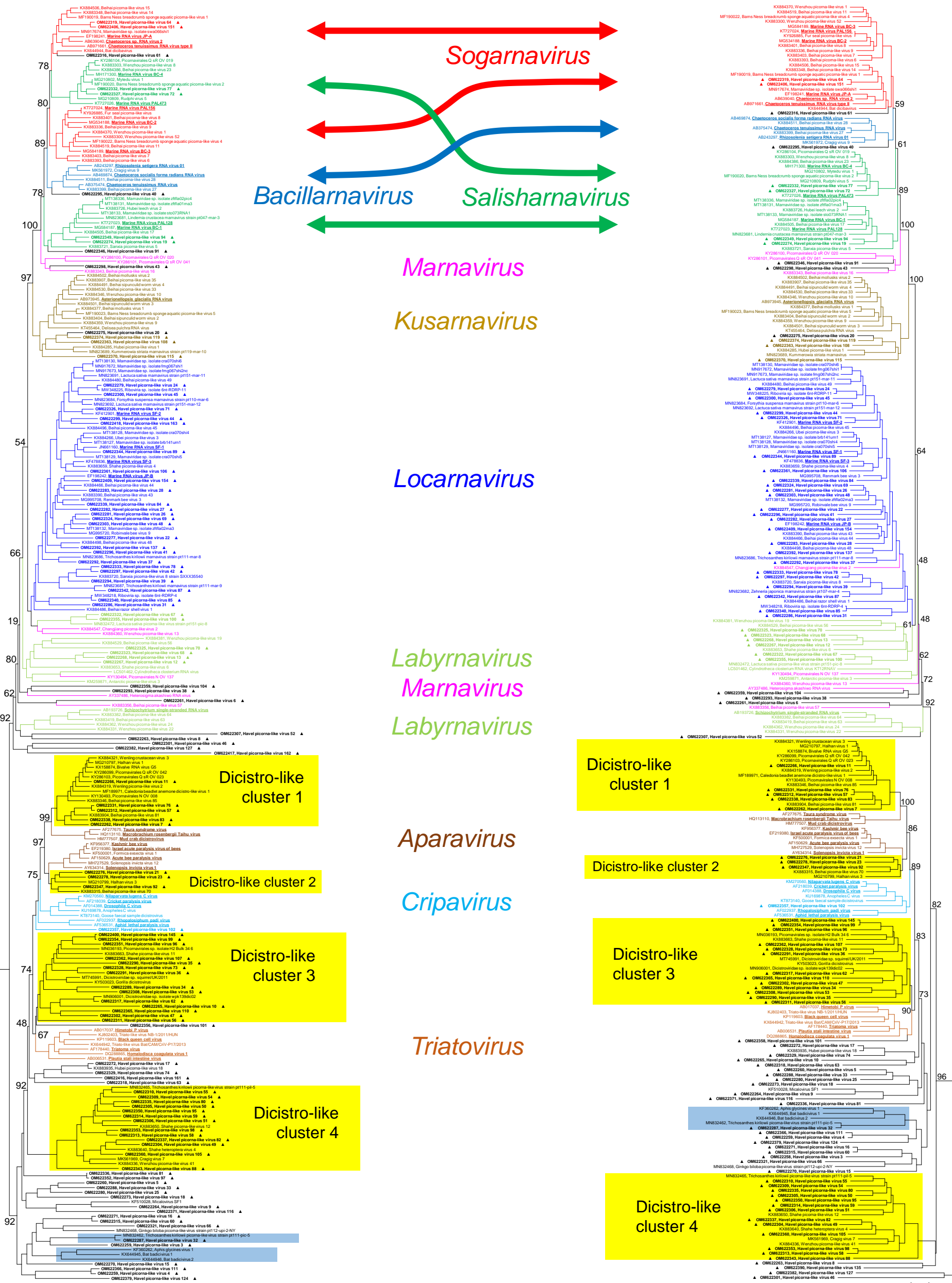

Supplement: Supplementary Figure 3 — Phylogenetic analysis of the VP1-to-helicase gene region of 15 acknowleged iflavirus strains, 20 candidate strains and Havel picorna-like viruses 14 and -129. The tree was inferred with IQ-Tree 2, optimal substitution model: GTR + F + R5. Numbers at nodes present bootstrap values obtained after 50,000 ultrafast bootstrap replications. The scale indicates substitutions per site. Presented are GenBank acc. nos. and virus names. Unassigned viruses are printed in blue. A triangle (▲) indicates the viruses of the present study. [file Data_Sheet_3.PDF]

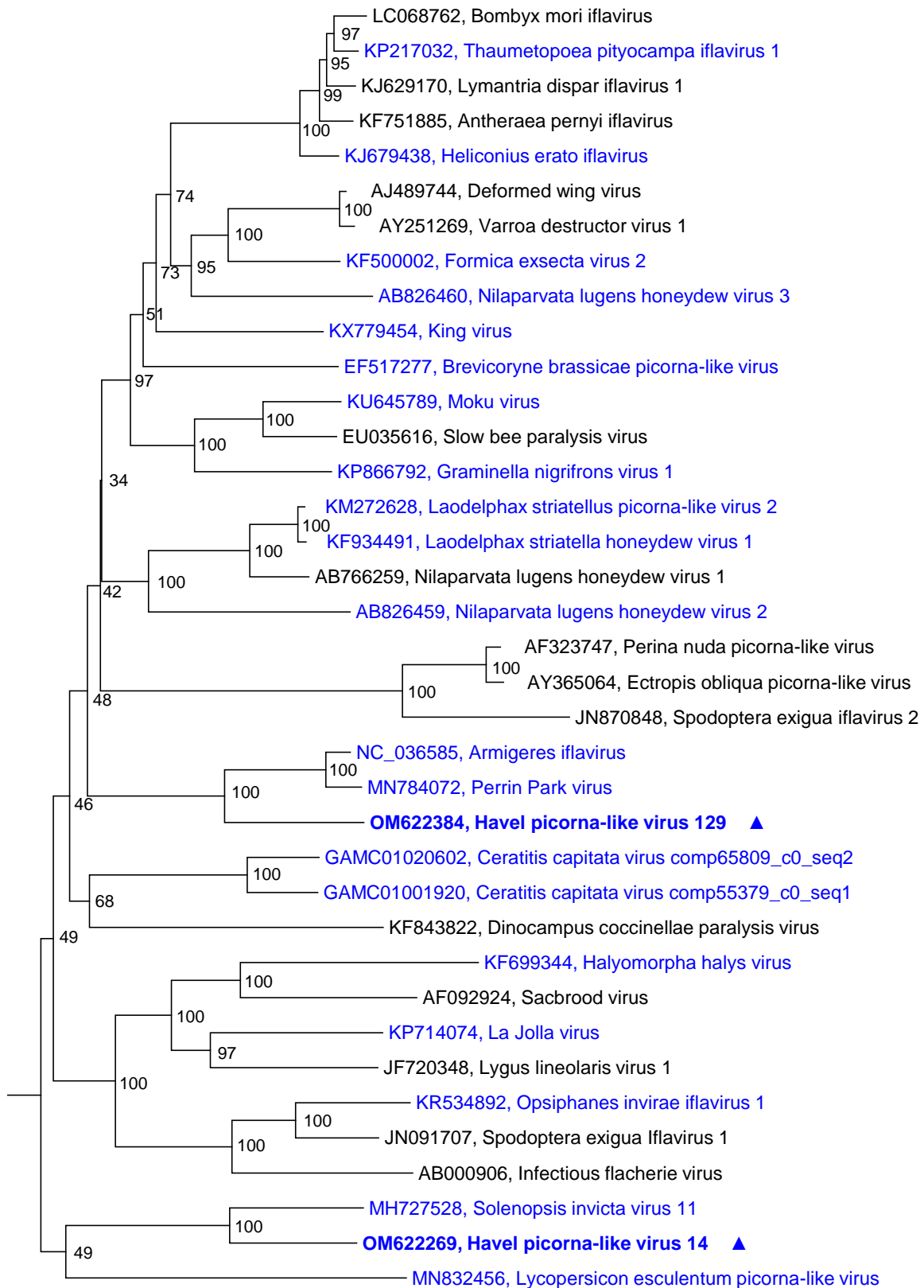

0.5

Supplement: Supplementary file 5 [file Data_Sheet_5.PDF]

B

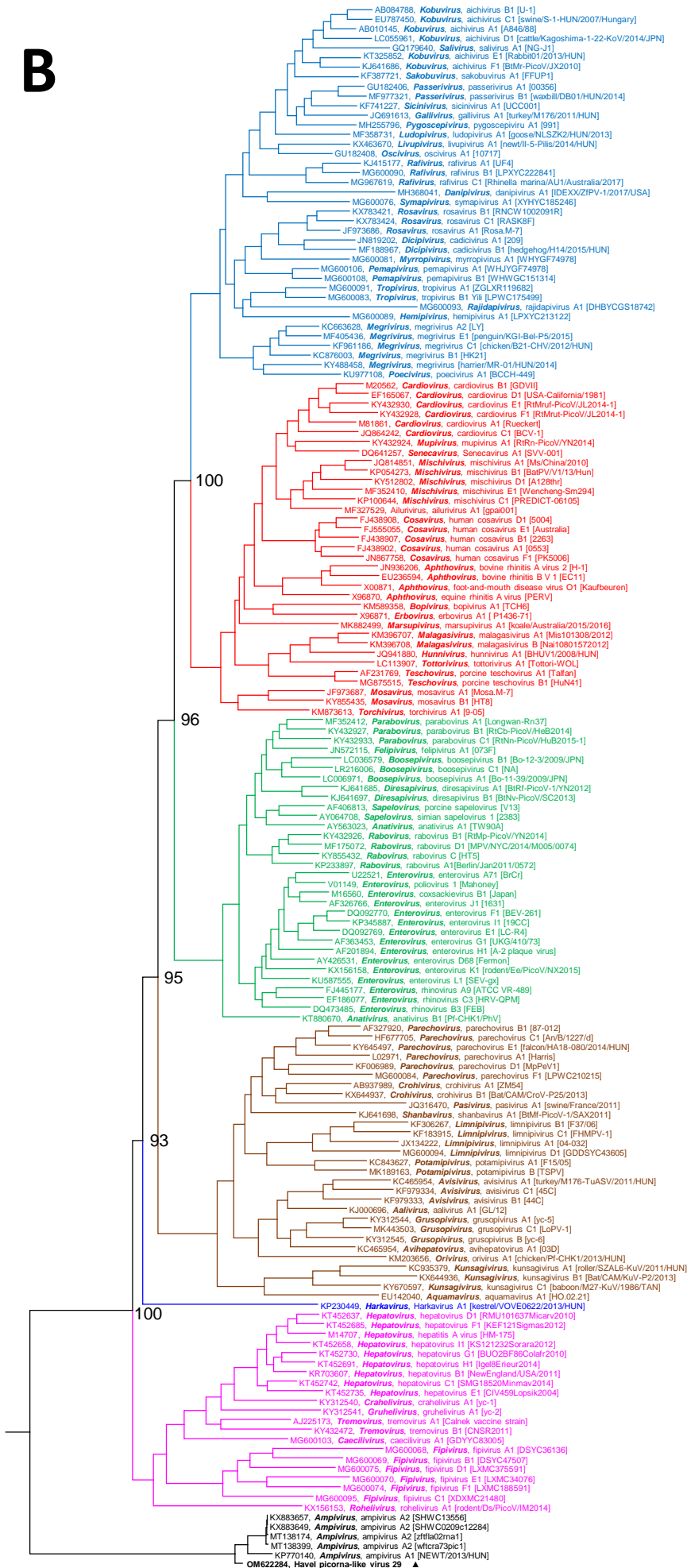

Kodimmesavirinae

Caphthovirinae

Ensavirinae

Paavivirinae

Heptrevirinae

0.5

Supplement: Supplementary file 7 [file Data_Sheet_7.PDF]
