# Supplementary material for: Picorna-Like Viruses of the Havel River, Germany
Source: Front Microbiol. 2022 Apr 4;13:865287. doi: 10.3389/fmicb.2022.865287 (PMC9013969; doi:10.3389/fmicb.2022.865287)
Supplement: Supplementary Figure 4 — (A) Phylogenetic analysis of the capsid protein-encoding gene region (P1) of Havel picorna-like virus 29 and 162 picornaviruses. The tree was inferred with IQ-Tree 2, optimal substitution model: GTR+F+R9. Presented are GenBank acc. nos., genera (printed in italics and bold), virus names and strain designations (in square brackets). Sub-family names where available are given to the right. A triangle (▲) indicates the virus of the present study. Numbers at nodes present bootstrap values obtained after 50,000 ultrafast bootstrap replications. The scale indicates substitutions per site. (B) Phylogenetic analysis of the proteinase/polymerase-encoding gene region (3CD) of Havel picorna-like virus 29 and 162 picornaviruses. The tree was inferred with IQ-Tree 2, optimal substitution model: GTR+F+R8. Presented are GenBank acc. nos., genera (printed in italics and bold), virus names and strain designations (in square brackets). Sub-family names where available are given to the right. A triangle (▲) indicates the virus of the present study. Numbers at nodes present bootstrap values obtained after 50,000 ultrafast bootstrap replications. The scale indicates substitutions per site. [file Data_Sheet_4.PDF]

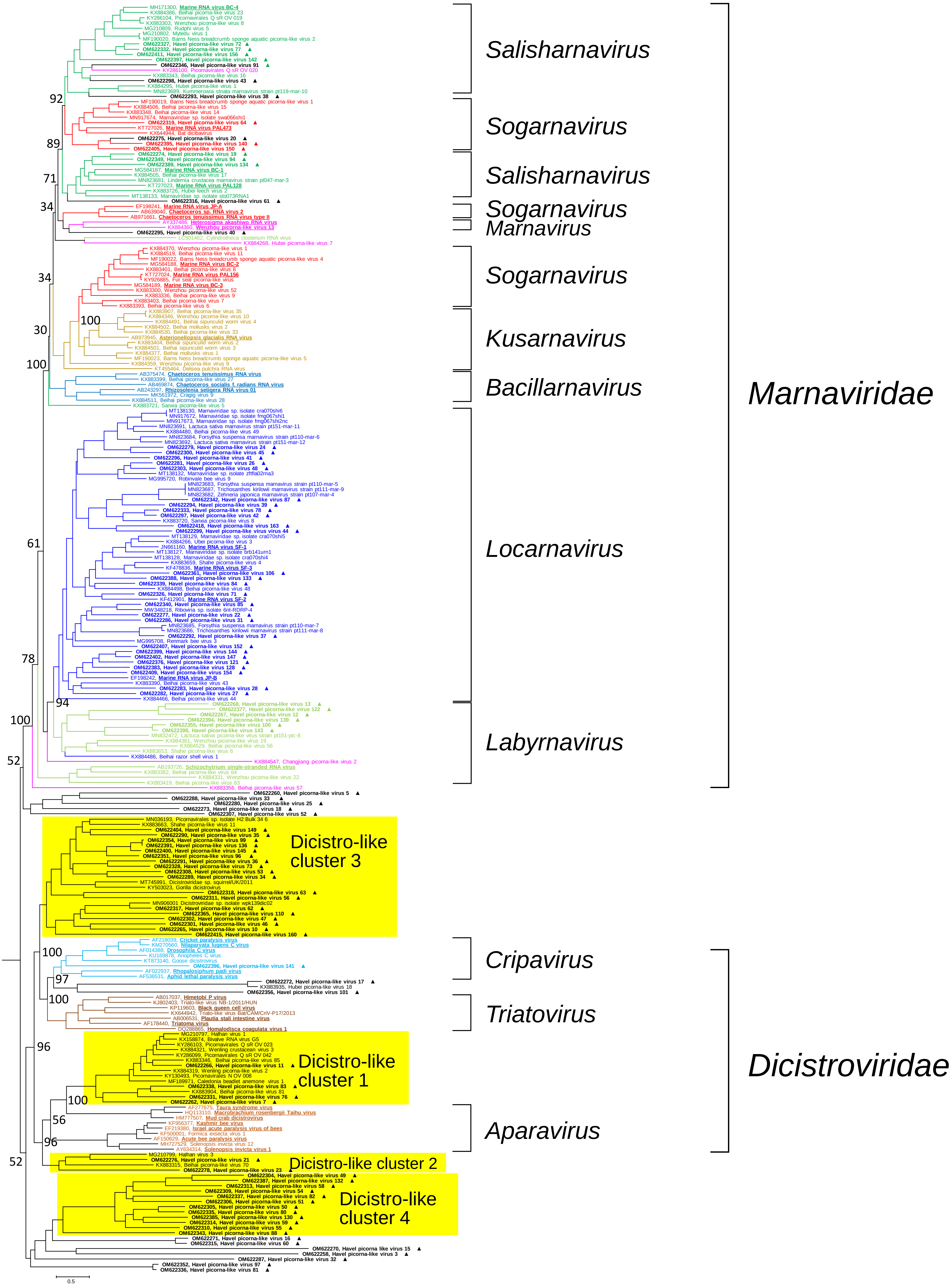

Salisharnavirus
Sogarnavirus
Salisharnavirus
Sogarnavirus
Marnavirus
Sogarnavirus
Kusarnavirus
Bacillarnavirus
Locarnavirus
Labyrnavirus
Marnaviridae
Dicistro-like cluster 3
Cripavirus
Triatovirus
Dicistro-like cluster 1
Aparavirus
Dicistro-like cluster 2
Dicistro-like cluster 4
Dicistroviridae
92
89
71
34
34
30
100
100
61
78
94
100
52
100
97
100
96
100
56
96
52
0.5
