# Supplementary material for: Picorna-Like Viruses of the Havel River, Germany
Source: Front Microbiol. 2022 Apr 4;13:865287. doi: 10.3389/fmicb.2022.865287 (PMC9013969; doi:10.3389/fmicb.2022.865287)
Supplement: Supplementary file 6 [file Data_Sheet_6.PDF]

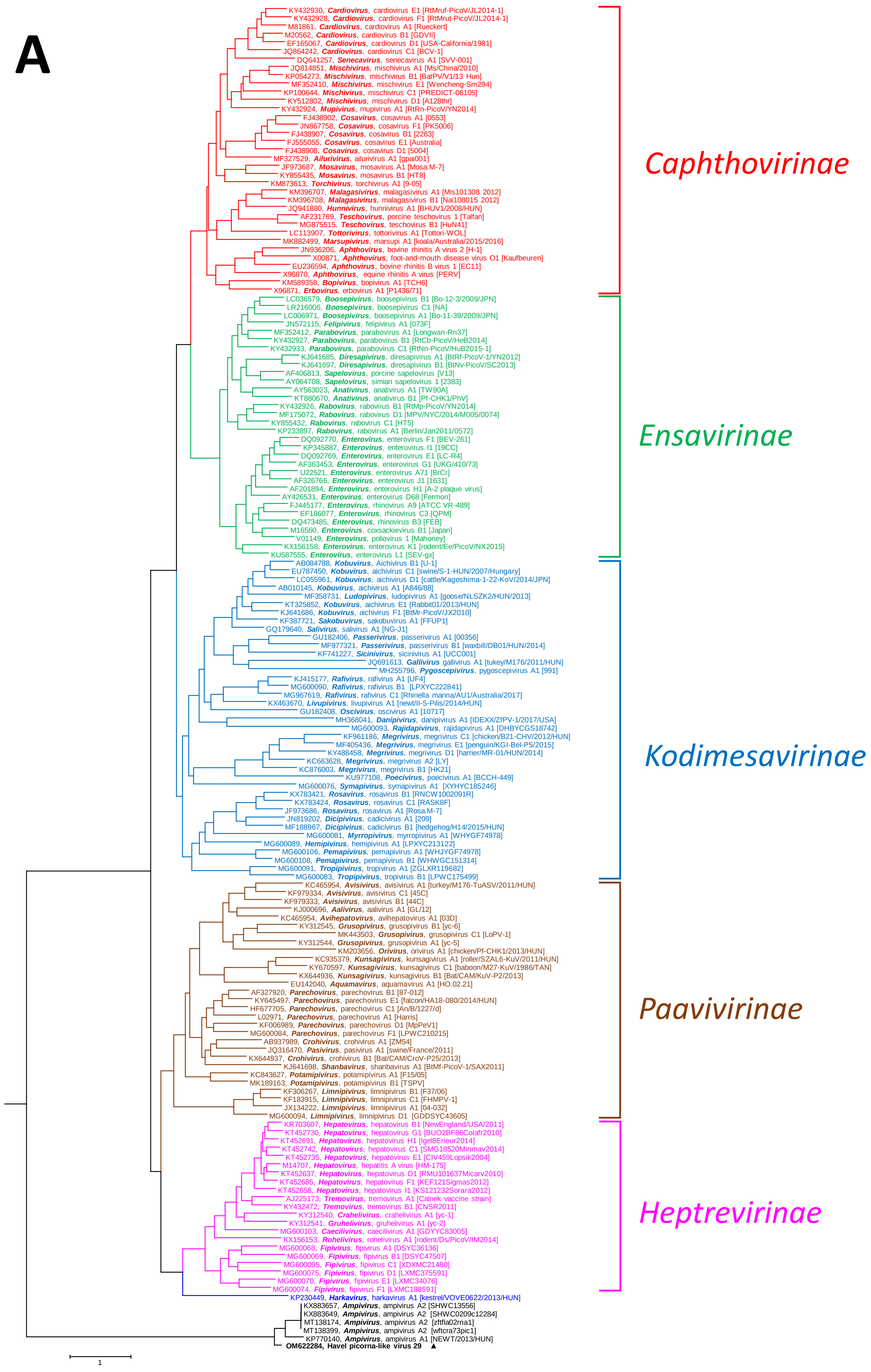
A
KY432930, Cardiovirus, cardiovirus E1 [RtMruf-PicoV/JL2014-1]
KY432928, Cardiovirus, cardiovirus F1 [RtMrut-PicoV/JL2014-1]
M81861, Cardiovirus, cardiovirus A1 [Rueckert]
M20562, Cardiovirus, cardiovirus B1 [GDVII]
EF165067, Cardiovirus, cardiovirus D1 [USA-California/1981]
JQ864242, Cardiovirus, cardiovirus C1 [BCV-1]
DQ641257, Senecavirus, senecavirus A1 [SVV-001]
JQ814851, Mischivirus, mischivirus A1 [Ms/China/2010]
KP054273, Mischivirus, mischivirus B1 [BatPV/V1/13 Hun]
MF352410, Mischivirus, mischivirus E1 [Wencheng-Sm294]
KP100644, Mischivirus, mischivirus C1 [PREDICT-06105]
KY512802, Mischivirus, mischivirus D1 [A128thr]
KY432924, Mupivirus, mupivirus A1 [RtRn-PicoV/YN2014]
FJ438902, Cosavirus, cosavirus A1 [0553]
JN867758, Cosavirus, cosavirus F1 [PK5006]
FJ438907, Cosavirus, cosavirus B1 [2263]
FJ555055, Cosavirus, cosavirus E1 [Australia]
FJ438908, Cosavirus, cosavirus D1 [5004]
MF327529, Ailurivirus, ailurivirus A1 [gpai001]
JF973687, Mosavirus, mosavirus A1 [Mosa.M-7]
KY855435, Mosavirus, mosavirus B1 [HT8]
KM873613, Torchivirus, torchivirus A1 [9-05]
KM396707, Malagasivirus, malagasivirus A1 [Mis101308 2012]
KM396708, Malagasivirus, malagasivirus B1 [Nai108015 2012]
JQ941880, Hunnivirus, hunnivirus A1 [BHUV1/2008/HUN]
AF231769, Teschovirus, porcine teschovirus 1 [Talfan]
MG875515, Teschovirus, teschovirus B1 [HuN41]
LC113907, Tottorivirus, tottorivirus A1 [Tottori-WOL]
MK882499, Marsupivirus, marsupi A1 [koala/Australia/2015/2016]
JN936206, Aphthovirus, bovine rhinitis A virus 2 [H-1]
X00871, Aphthovirus, foot-and-mouth disease virus O1 [Kaufbeuren]
EU236594, Aphthovirus, bovine rhinitis B virus 1 [EC11]
X96870, Aphthovirus, equine rhinitis A virus [PERV]
KM589358, Bopivirus, bopivirus A1 [TCH6]
X96871, Erbovirus, erbovirus A1 [P1436/71]
LC036579, Boosepivirus, boosepivirus B1 [Bo-12-3/2009/JPN]
LR216006, Boosepivirus, boosepivirus C1 [NA]
LC006971, Boosepivirus, boosepivirus A1 [Bo-11-39/2009/JPN]
JN572115, Felipivirus, felipivirus A1 [073F]
MF352412, Parabovirus, parabovirus A1 [Longwan-Rn37]
KY432927, Parabovirus, parabovirus B1 [RtCb-PicoV/HeB2014]
KY432933, Parabovirus, parabovirus C1 [RtNn-PicoV/HuB2015-1]
KJ641685, Diresapivirus, diresapivirus A1 [BtRf-PicoV-1/YN2012]
KJ641697, Diresapivirus, diresapivirus B1 [BtNv-PicoV/SC2013]
AF406813, Sapelovirus, porcine sapelovirus [V13]
AY064708, Sapelovirus, simian sapelovirus 1 [2383]
AY563023, Anativirus, anativirus A1 [TW90A]
KT880670, Anativirus, anativirus B1 [Pf-CHK1/PhV]
KY432926, Rabovirus, rabovirus B1 [RtMp-PicoV/YN2014]
MF175072, Rabovirus, rabovirus D1 [MPV/NYC/2014/M005/0074]
KY855432, Rabovirus, rabovirus C1 [HT5]
KP233897, Rabovirus, rabovirus A1 [Berlin/Jan2011/0572]
DQ092770, Enterovirus, enterovirus F1 [BEV-261]
KP345887, Enterovirus, enterovirus I1 [19CC]
DQ092769, Enterovirus, enterovirus E1 [LC-R4]
AF363453, Enterovirus, enterovirus G1 [UKG/410/73]
U22521, Enterovirus, enterovirus A71 [BrCr]
AF326766, Enterovirus, enterovirus J1 [1631]
AF201894, Enterovirus, enterovirus H1 [A-2 plaque virus]
AY426531, Enterovirus, enterovirus D68 [Fermon]
FJ445177, Enterovirus, rhinovirus A9 [ATCC VR-489]
EF186077, Enterovirus, rhinovirus C3 [QPM]
DQ473485, Enterovirus, rhinovirus B3 [FEB]
M16560, Enterovirus, coxsackievirus B1 [Japan]
V01149, Enterovirus, poliovirus 1 [Mahoney]
KX156158, Enterovirus, enterovirus K1 [rodent/Ee/PicoV/NX2015]
KU587555, Enterovirus, enterovirus L1 [SEV-gx]
AB084788, Kobuvirus, Aichivirus B1 [U-1]
EU787450, Kobuvirus, aichivirus C1 [swine/S-1-HUN/2007/Hungary]
LC055961, Kobuvirus, aichivirus D1 [cattle/Kagoshima-1-22-KoV/2014/JPN]
AB010145, Kobuvirus, aichivirus A1 [A846/88]
MF358731, Ludopivirus, ludopivirus A1 [goose/NLSZK2/HUN/2013]
KT325852, Kobuvirus, aichivirus E1 [Rabbit01/2013/HUN]
KJ641686, Kobuvirus, aichivirus F1 [BtMr-PicoV/JX2010]
KF387721, Sakobuvirus, sakobuvirus A1 [FFUP1]
GQ179640, Salivirus, salivirus A1 [NG-J1]
GU182406, Passerivirus, passerivirus A1 [00356]
MF977321, Passerivirus, passerivirus B1 [waxbill/DB01/HUN/2014]
KF741227, Sicinivirus, sicinivirus A1 [UCC001]
JQ691613, Gallivirus gallivirus A1 [tukey/M176/2011/HUN]
MH255796, Pygoscepivirus, pygoscepivirus A1 [991]
KJ415177, Rafivirus, rafivirus A1 [UF4]
MG600090, Rafivirus, rafivirus B1 [LPXYC222841]
MG967619, Rafivirus, rafivirus C1 [Rhinella marina/AU1/Australia/2017]
KX463670, Livupivirus, livupivirus A1 [newt/II-5-Pilis/2014/HUN]
GU182408, Oscivirus, oscivirus A1 [10717]
MH368041, Danipivirus, danipivirus A1 [IDEXX/ZfPV-1/2017/USA]
MG600093, Rajidapivirus, rajidapivirus A1 [DHBYCGS18742]
KF961186, Megrivirus, megrivirus C1 [chicken/B21-CHV/2012/HUN]
MF405436, Megrivirus, megrivirus E1 [penguin/KGI-Bel-P5/2015]
KY488458, Megrivirus, megrivirus D1 [harrier/MR-01/HUN/2014]
KC663628, Megrivirus, megrivirus A2 [LY]
KC876003, Megrivirus, megrivirus B1 [HK21]
KU977108, Poecivirus, poecivirus A1 [BCCH-449]
MG600076, Symapivirus, symapivirus A1 [XYHYC185246]
KX783421, Rosavirus, rosavirus B1 [RNCW1002091R]
KX783424, Rosavirus, rosavirus C1 [RASK8F]
JF973686, Rosavirus, rosavirus A1 [Rosa.M-7]
JN819202, Dicipivirus, cadicivirus A1 [209]
MF188967, Dicipivirus, cadicivirus B1 [hedgehog/H14/2015/HUN]
MG600081, Myrropivirus, myrropivirus A1 [WHYGF74978]
MG600089, Hemipivirus, hemipivirus A1 [LPXYC213122]
MG600106, Pemapivirus, pemapivirus A1 [WHJYGF74978]
MG600108, Pemapivirus, pemapivirus B1 [WHWGC151314]
MG600091, Tropivirus, tropivirus A1 [ZGLXR119682]
MG600083, Tropivirus, tropivirus B1 [LPWC175499]
KC465954, Avisivirus, avisivirus A1 [turkey/M176-TuASV/2011/HUN]
KF979334, Avisivirus, avisivirus C1 [45C]
KF979333, Avisivirus, avisivirus B1 [44C]
KJ000696, Aalivirus, aalivirus A1 [GL/12]
KC465954, Avihepatovirus, avihepatovirus A1 [03D]
KY312545, Grusopivirus, grusopivirus B1 [yc-6]
MK443503, Grusopivirus, grusopivirus C1 [LoPV-1]
KY312544, Grusopivirus, grusopivirus A1 [yc-5]
KM203656, Orivirus, orivirus A1 [chicken/Pf-CHK1/2013/HUN]
KC935379, Kunsagivirus, kunsagivirus A1 [roller/SZAL6-KuV/2011/HUN]
KY670597, Kunsagivirus, kunsagivirus C1 [baboon/M27-KuV/1986/TAN]
KX644936, Kunsagivirus, kunsagivirus B1 [Bat/CAM/KuV-P2/2013]
EU142040, Aquamavirus, aquamavirus A1 [HO.02.21]
AF327920, Parechovirus, parechovirus B1 [87-012]
KY645497, Parechovirus, parechovirus E1 [falcon/HA18-080/2014/HUN]
HF677705, Parechovirus, parechovirus C1 [An/B/1227/d]
L02971, Parechovirus, parechovirus A1 [Harris]
KF006989, Parechovirus, parechovirus D1 [MpPeV1]
MG600084, Parechovirus, parechovirus F1 [LPWC210215]
AB937989, Crohivirus, crohivirus A1 [ZM54]
JQ316470, Pasivirus, pasivirus A1 [swine/France/2011]
KX644937, Crohivirus, crohivirus B1 [Bat/CAM/CroV-P25/2013]
KJ641698, Shanbavirus, shanbavirus A1 [BtMf-PicoV-1/SAX2011]
KC843627, Potamipivirus, potamipivirus A1 [F15/05]
MK189163, Potamipivirus, potamipivirus B1 [TSPV]
KF306267, Limnipivirus, limnipivirus B1 [F37/06]
KF183915, Limnipivirus, limnipivirus C1 [FHMPV-1]
JX134222, Limnipivirus, limnipivirus A1 [04-032]
MG600094, Limnipivirus, limnipivirus D1 [GDDSYC43605]
KR703607, Hepatovirus, hepatovirus B1 [NewEngland/USA/2011]
KT452730, Hepatovirus, hepatovirus G1 [BUO2BF86Colafr2010]
KT452691, Hepatovirus, hepatovirus H1 [Igel8Erieur2014]
KT452742, Hepatovirus, hepatovirus C1 [SMG18520Minmav2014]
KT452735, Hepatovirus, hepatovirus E1 [CIV459Lopsik2004]
M14707, Hepatovirus, hepatitis A virus [HM-175]
KT452637, Hepatovirus, hepatovirus D1 [RMU101637Micarv2010]
KT452685, Hepatovirus, hepatovirus F1 [KEF121Sigmas2012]
KT452658, Hepatovirus, hepatovirus I1 [KS121232Sorara2012]
AJ225173, Tremovirus, tremovirus A1 [Calnek vaccine strain]
KY432472, Tremovirus, tremovirus B1 [CNSR2011]
KY312540, Crahelivirus, crahelivirus A1 [yc-1]
KY312541, Gruhelivirus, gruhelivirus A1 [yc-2]
MG600103, Caecilivirus, caecilivirus A1 [GDYYC83005]
KX156153, Rohelivirus, rohelivirus A1 [rodent/Ds/PicoV/IM2014]
MG600068, Fipivirus, fipivirus A1 [DSYC36136]
MG600069, Fipivirus, fipivirus B1 [DSYC47507]
MG600095, Fipivirus, fipivirus C1 [XDXMC21480]
MG600075, Fipivirus, fipivirus D1 [LXMC375591]
MG600070, Fipivirus, fipivirus E1 [LXMC34076]
MG600074, Fipivirus, fipivirus F1 [LXMC188591]
KP230449, Harkavirus, harkavirus A1 [kestrel/VOVE0622/2013/HUN]
KX883657, Ampivirus, ampivirus A2 [SHWC13556]
KX883649, Ampivirus, ampivirus A2 [SHWC0209c12284]
MT138174, Ampivirus, ampivirus A2 [zftfla02rna1]
MT138399, Ampivirus, ampivirus A2 [wftcra73pic1]
KP770140, Ampivirus, ampivirus A1 [NEWT/2013/HUN]
OM622284, Havel picorna-like virus 29 ▲
1
Caphthovirinae
Ensavirinae
Kodimesavirinae
Paavivirinae
Heptrevirinae
